# Occlusion body-incorporated SARS-CoV-2 S-RBD via cypovirus polyhedrin-derived peptide: a novel insect cell-expressed antigen for high-accuracy COVID-19 diagnosis, and humoral immune response and hybrid immunity evaluation

**DOI:** 10.1590/S1678-9946202668012

**Published:** 2026-02-16

**Authors:** Murilo Barros-Silveira, Fabricio da Silva Morgado, Laurine Lacerda Pigosso, Fernanda Cortez Roriz Pontes, Ethiane Rozo dos Santos, Rosana Pereira Morais, Yves Mauro Fernandes Ternes, Célia Maria de Almeida Soares, Bergmann Morais Ribeiro, Fátima Ribeiro-Dias

**Affiliations:** 1 Universidade Federal de Goiás, Instituto de Patologia Tropical e Saúde Pública, Laboratório de Imunidade Natural, Goiânia, Goiás, Brazil; 2 Universidade de Brasília, Instituto de Ciências Biológicas, Departamento de Biologia Celular, Laboratório de Baculovírus, Distrito Federal, Brazil; 3 Universidade Federal de Goiás, Instituto de Ciências Biológicas II, Goiânia, Goiás, Brazil

**Keywords:** Serological assay, ELISA, Pandemic, S-RBD-POLH, Vaccine

## Abstract

Accurate serological assays are critical for monitoring antibody responses to SARS-CoV-2 and for assessing vaccine efficacy. In this study, we engineered a recombinant baculovirus to express the SARS-CoV-2 spike receptor-binding domain (RBD) fused to an N-terminal peptide of cypovirus polyhedrin (S-RBD-POLH), thereby enabling its potential encapsulation within occlusion bodies. The construct was expressed in insect cells and employed to establish an in-house enzyme-linked immunosorbent assay (ELISA-S) for COVID-19 diagnosis and evaluation of the humoral immune response to SARS-CoV-2. We compared the performance of ELISA-S with a commercial lateral flow immunoassay (LFIA), a chemiluminescence immunoassay (CLIA), and an in-house ELISA targeting *Escherichia coli*-produced nucleocapsid (N) protein (ELISA-N). Both in-house ELISAs had excellent diagnostic performance (overall accuracy = 0.90), with ELISA-S achieving 98.9% sensitivity and 100% specificity. Inter-assay concordance was high (kappa coefficient > 0.92), with near-perfect agreement between ELISA-S and LFIA (kappa = 0.99). Longitudinal analysis revealed increasing anti-RBD IgG titers over time, in contrast to declining anti-N IgG levels, consistent with expected post-infection kinetics. Moreover, ELISA-S reliably detected antibodies in individuals with hybrid immunity across different periods of the pandemic. These findings establish a scalable platform for antigen encapsulation and production in insect cells to enhance antibody detection. The ELISA-S system represents a robust and adaptable approach for evaluating humoral immunity following infection, vaccination, or hybrid immunity, and can be used for all variants.

## INTRODUCTION

COVID-19, caused by Severe Acute Respiratory Syndrome Coronavirus 2 (SARS-CoV-2)^1,2^, has led to more than 600 million confirmed cases and approximately seven million deaths worldwide since its emergence^2^. The viral spike (S) protein, a major surface antigen, mediates host cell entry through binding of its S1 subunit to the angiotensin-converting enzyme 2 (ACE2) receptor, while the S2 subunit drives membrane fusion. As a highly immunogenic target, the S protein elicits robust humoral and cellular immune responses, making it central to both therapeutic and vaccine development^3,4^. However, its pronounced mutability has facilitated the emergence of immune-evading variants with altered virulence, transmissibility, and resistance to neutralizing antibodies^4,5^, thereby complicating diagnosis and treatment.

In addition to the S protein, the nucleocapsid (N) protein represents another immunodominant antigen that is abundantly expressed during infection^5^. Beyond its essential role in viral replication, the N protein modulates host immune responses by inducing cytokine and chemokine production in lung cells^3^ and by eliciting strong antibody responses. Due to its relatively conserved sequence compared to the S protein, the N protein serves as a more stable diagnostic target, particularly during the acute phase of infection^5^. Given their complementary advantages, both S and N proteins have been widely employed in SARS-CoV-2 diagnostics^4,6^. Whereas S-based assays are indispensable for evaluating vaccine-induced immunity, N-based tests provide reliable markers of natural infection across different viral variants.

In Brazil, the Ministry of Health has approved multiple immunoassays for COVID-19 serological testing throughout the pandemic^6–10^, with performance extensively evaluated by national research groups^8–10^. These tests primarily target SARS-CoV-2 S and N proteins and are implemented via various platforms, including enzyme-linked immunosorbent assays (ELISA), lateral flow immunoassays (LFIA), and chemiluminescence immunoassays (CLIA). Comparative analyses have highlighted critical differences in performance. Cota *et al*.^9^ demonstrated that LFIAs generally showed higher specificity (59.5%–83.1%) than ELISAs (58.7%–76.8%), whereas ELISAs showed superior sensitivity (50.7%–92.6%). Cross-reactivity was reported with certain infections, including leishmaniasis and arboviruses, but not with chikungunya, HIV, dengue, or hepatitis B virus^9^. Similarly, Castejon *et al*.^8^ reported high specificity but low sensitivity for N protein-based LFIA and CLIA assays.

A comprehensive review by Vedova-Costa *et al*.^10^ emphasized that ELISA remains the gold standard for serological testing due to its reliability and scalability. While manufacturers report ELISA sensitivity ranging from 55% to 100% and specificity between 85% and 100%, real-world performance often deviates from these values, with accuracy strongly dependent on the selected antigen target. In Brazil, about 80% of COVID-19 diagnostic tests are imported^6,9^, underscoring the country's reliance on foreign technologies during public health crises. Early in the pandemic, most serological assays targeted either the full-length S protein or its S1 subunit containing the receptor-binding domain (RBD)^11–14^. Subsequently, N protein-based tests gained prominence, demonstrating improved specificity due to lower sequence variability and reduced cross-reactivity compared to S-based assays^13–15^.

To address the need for stable and high-yield SARS-CoV-2 antigens for diagnostic and immunological applications, we employed a baculovirus expression system leveraging the unique properties of cypovirus occlusion bodies (OBs). Cypoviruses (CPVs; *Spinareoviridae* family) are insect-specific pathogens that form protective OBs composed of polyhedrin (POLH) protein crystals. These OBs naturally encapsulate viral particles but can be repurposed to incorporate heterologous proteins via POLH-mediated assembly. The OB structure consists of POLH trimers organized into tetrahedral clusters stabilized by non-covalent interactions, particularly via the N-terminal α-helix 1 (H1) domain. Efficient incorporation in OBs can be achieved by fusing target proteins (e.g., S-RBD) to H1 and co-expressing them with full-length POLH^16,17^. This strategy provides three major advantages for diagnostic development: (i) enhanced stability, as OB encapsulation preserves protein conformation and functionality during storage; (ii) multiplexing capacity, enabling co-encapsulation of multiple antigens for multi-analyte detection^16^; and (iii) retention of bioactivity, with OB-embedded antigens maintaining immunoreactivity, as demonstrated for other pathogens^17^. Collectively, these features position POLH-based OBs as a versatile platform for generating robust, scalable diagnostic reagents capable of functioning across diverse settings.

This study aims to develop a stable, high-yield SARS-CoV-2 S-RBD antigen using an insect cell expression system, in both OB-incorporated and soluble forms, for COVID-19 serodiagnosis. We established two in-house ELISA platforms: one employing this recombinant S-RBD-POLH (ELISA-S) and another utilizing *E. coli*-produced N protein (ELISA-N). To validate this approach, we systematically compared these assays with two commercial tests widely deployed in Brazil during the pandemic. Assay performance was evaluated across diverse immunological contexts, including primary SARS-CoV-2 infection, vaccine-induced immunity, and hybrid immunity (infection plus vaccination) during different stages of the pandemic. Our findings provide critical insights into large-scale serological testing in Brazil and contribute to a deeper understanding of SARS-CoV-2 humoral immunity dynamics, while also addressing the urgent need for reliable, locally produced diagnostic reagents to strengthen national pandemic preparedness.

### Ethics

The study protocol was approved by the Ethics Committee of Hospital das Clinicas (CAAE N° 32950720.0.0000.5078). All participants provided written informed consent.

## MATERIALS AND METHODS

### Samples

First cohort: a convenience sample was obtained from employees of the Goiania Municipality Health Department (Goias State, Brazil). The study cohort comprised two groups: (i) 200 individuals without reported COVID-19 and (ii) 200 individuals with self-reported symptoms of the disease. Participants were consecutively enrolled during visits for COVID-19 testing, at which blood was collected for serological analysis.

Most participants (n = 300) were enrolled between June and July 2020, with an additional 100 participants enrolled from January to April 2021, yielding a total of 400. Symptomatic individuals were stratified by clinical presentation into mild cases (e.g., fever, headache, and myalgia) or moderate cases (e.g., memory loss, dyspnea). Among the 200 symptomatic individuals, SARS-CoV-2 infection was confirmed in 175 (87.5%) via RT-qPCR. Nasopharyngeal swabs were analyzed at the Central Laboratory of Public Health (LACEN), Goias State, using the Bio-Manguinhos (Fiocruz-RJ, Brazil) approved protocol. Overall, 181 of the 400 individuals tested negative by RT-qPCR (Supplementary Figure S1A). Only one serum sample was obtained from each participant (n = 400 serum samples).

During the period of study (2020–2021) circulating variants of concern (VOCs) in Goias included Alpha (B.1.1.7), Beta (B.1.351), Gamma (P.1), and Delta (B.1.617)^18^. A pre-pandemic control group (n = 47) consisted of sera collected in 2019, serving as true negatives to confirm the absence of SARS-CoV-2 antibodies. The cohort (Supplementary Figure S1A) was used to evaluate the diagnostic performance of the ELISAs for anti-SARS-CoV-2 antibody detection as well as humoral immune response dynamics.

A second cohort was assembled to evaluate hybrid immunity (natural infection plus vaccination), which included 58 individuals immunized with two doses of ChAdOx1 nCoV-19/AstraZeneca (Fiocruz/Covishield, batches CTNAV520 and 214VCD046W) between 2021 and 2022. Participants were stratified into: (i) Post-COVID-19 group (n = 17; most of them reported previous COVID-19 six to eight months (or more) before), with prior RT-qPCR-confirmed infection; and (ii) Infection-naïve group (n = 41), with no history of infection. Prior infection status and antibody profiles were confirmed using the LABScreen™ COVID Plus assay (OneLambda, Canoga Park, CA, USA), which includes antigens from seasonal coronaviruses, SARS-CoV, and MERS-CoV, enabling cross-reactivity assessment. None of the sera tested positive for non-SARS-CoV-2 antigens. Blood samples were collected at three time points: baseline (prior to the first vaccine dose), 30 days after the first dose, and 30 days after the second dose. The interval between doses was one month. During this period (2021–2022), circulating VOCs in Goias included Alpha (B.1.1.7), Gamma (P.1), and Delta (B.1.617.2)^18,19^. Supplementary Figure S1B summarizes the study design.

The third cohort was designed to assess breakthrough infections in vaccinated individuals, which consisted of 40 sera collected in 2023–2024 from individuals with RT-qPCR-confirmed COVID-19 (Bio-Manguinhos protocol). All had received one of the available vaccine regimens in Goias: CoronaVac (Sinovac Biotech), ChAdOx1 nCoV-19 (AstraZeneca), Ad26.COV2.S/JNJ-78436735 (Janssen), or Tozinameran/Comirnaty (Pfizer-BioNTech). Predominant circulating variants of concern (VOCs) during this period included Alpha (B.1.1.7), Gamma (P.1), and Delta (B.1.617.2), with subsequent predominance of Omicron (BQ.1.1) and the Omicron subvariant EG.5^19^. This cohort represents a model of hybrid immunity generated by vaccination followed by infection (Supplementary Figure S1C).

### Serological screening: lateral flow immunoassay (LFIA) for detection of anti-S antibodies

Anti-SARS-CoV-2 S protein IgM/IgG antibodies were detected using a lateral flow immunochromatographic assay (LFIA; SARS-CoV-2 Antibody Test^®^, Wondfo, Beijing, China). A total of 400 blood samples were collected via venipuncture (4 mL per participant) into serum-separator tubes (BD, New Jersey, USA), centrifuged at 3,500 × g for 15 min, and serum tested following the manufacturer's protocol. Samples were classified as reactive or non-reactive. The test was used during a mass epidemiological survey in Goiania city. Reported performance: sensitivity 86.43% (95%CI: 82.51–89.58%) and specificity 99.57% (95%CI: 97.63–99.92%).

### Chemiluminescence immunoassay (CLIA) for anti-N antibodies

Sera from 400 individuals were tested for IgG antibodies to the N protein using Abbott Architect/Alinity SARS-CoV-2 IgG CLIA (ARCHITECT™ i2000SR, Abbott Diagnostics, Abbott Park, IL, USA). This semi-quantitative assay follows manufacturer's instructions. Chemiluminescence is measured as relative light units (RLU), and result is expressed as a calculated index (S/C), which > 0.5 is considered positive (reagent serum). Reported sensitivity and specificity were 96.7% and 98.9%, respectively.

### Production of recombinant S-RBD protein with poyhedrin (S-RBD-POLH)

Supplementary File S1 shows a detailed description. Briefly, the gene encoding the receptor-binding domain of the SARS-CoV-2 spike protein was amplified by RT-PCR from a clinical sample^6^ and cloned into a modified pFastBac^™^ Dual plasmid (Thermofischer Scientific, Massachusetts, USA), under the control of the Autographa californica multiple nucleopolyhedrovirus (AcMNPV)'s polyhedrin promoter. This construct, designed to fuse the S-RDB with a segment of the *Thyrinteina arnobia* cypovirus 14 (TharCPV-14) *polyhedrin* gene^15,17,20^, was used to generate a recombinant baculovirus (BACTharCPVPOLHS-RBD) using the Bac-to-Bac technology (Thermofischer Scientific, Massachusetts, USA). A second recombinant baculovirus was constructed without a copy of the TharCPV *polyhedrin* gene using the same methodology and called BACS-RBD. The resulting baculoviruses were used to infect *Spodoptera cosmioides* larvae to produce cytoplasmic occlusion bodies (OBs) containing the S-RBD antigen.

For protein production, fourth-instar larvae were inoculated with the recombinant baculoviruses. At five days post-infection (dpi), OBs were extracted and purified from the larval homogenate. Supplementary File S1 provides detailed infection, homogenization, and centrifugation-based purification steps. The purified OBs were analyzed by SDS-PAGE and Western blot (Supplementary Figure S2), and the total protein concentration was quantified using the BCA method (Pierce BCA Protein Assay Kit, Thermo Scientific, Rockford, USA).

### Production of the recombinant N protein

The gene encoding N-CV19 cloned into the expression vector pET-28a was synthesized by GenOne Biotechnologies (Rio de Janeiro, Brazil). Bacterial cells, strain *Escherichia coli* BL21, harboring the recombinant plasmid were grown in Luria–Bertani (LB) medium supplemented with 50 μg/mL kanamycin (w/v) under agitation at 37 °C until the optical density (OD) reached an absorbance of 0.6 at a wavelength of 600 nm. The reagent Isopropyl-β-D-thiogalactopyranoside (IPTG) was added to the growing culture to a final concentration of 0.1 mM. The bacterial cells were harvested by centrifugation at 10,000 × *g* for 10 min after 6 h of incubation at 37 °C and resuspended in phosphate buffered saline (PBS) 1X. The protein was solubilized using 250 μL of Lysozyme (10 mg/ml) for 1.5 h. After, 50 μL of a 20% (w/v) N-lauroylsarcosine sodium salt (Sigma Aldrich, Missouri, KS, USA) solution were added for 5 mL of bacterial extracts and sonicated (five times, for 10 min). SDS-PAGE analysis showed the protein in the soluble fraction, and then the protein was purified by a nickel resin chromatography system (Qiagen Inc., Germantown, MD, USA). Protein was quantified using the BCA method (Pierce BCA Protein Assay Kit).

### In-house enzyme-linked immunosorbent assays for detection of IgG antibodies to SARS-CoV-2 proteins

The S-RBD antigen with (S-RBD-POLH) or without (S-RBD) polyhedrin was tested at 4 μg/mL, 2 μg/mL, and 1 μg/mL, based on prior protocols^21–23^. For the in-house ELISA-N, the N protein was tested at 5 μg/mL, 3 μg/mL, and 2 μg/mL^22^. The proteins were diluted into 0.1 M sodium carbonate-sodium bicarbonate buffer, pH 9.6 and distributed (50 μL/well) on 96 six-flat-bottomed polystyrene plates with high binding capacity (Corning Incorporated Costar^®^, Brazil). The plates were incubated at 4 °C for approximately 18 h. For the blocking step, a 0.1 M sodium carbonate-sodium bicarbonate buffer solution, pH 9.6, containing 5% skimmed milk powder and 1% bovine serum albumin (BSA, A3059-50G, Sigma-Aldrich, Missouri, USA) was used. After the incubation period with the antigens, the excess was discarded and three washes were performed with PBS washing solution containing 0.02% Tween 20 (PBS-T). The plates were incubated with 200 μL of blocking solution/well for 2 h, at room temperature (~21 °C, r.t.). Then, the wells were washed three times with PBS-T.

After, 50 μL of each reactive serum, RS (47 sera were randomly selected from RT-qPCR positive tests, and presence of antibodies confirmed by LFIA) or non-reactive serum, NRS (47 sera from healthy individuals collected in 2019, before the pandemics, and negative in LFIA) were diluted 1:50-1:400 in PBS containing 5% skimmed milk powder and 0.1% BSA (sample diluent solution). The plates were incubated for 1 h (r.t.), followed by washings (3x). For detection of IgG against S-RBD-POLH protein or N protein, the conjugate anti-human IgG antibodies (H+L)-peroxidase (HRP; Life Technologies, California, USA) was diluted to 1:500-1:2000 (starting as indicated by the manufacturer), 50 μL per well. The plates were incubated for 1 h (r.t.) and then washed eight times. The substrate/chromogenic solution Tetramethylbenzidine (TMB) (Life Technologies, California, USA) was added (50 μL) to each well, 10 min (r.t.), protected from the light. Then, 20 μL of 2 N sulfuric acid solution was added to stop the enzymatic reaction. Absorbances were obtained at 450 nm with a reference filter at 620 nm, using a Multiskan^TM^ microplate spectrophotometer (Thermo Fisher ScientificTM, Massachusetts, USA).

For this standardization phase, RS (n = 47 patients, PT) and NRS or controls (n = 47 controls, CT) were analyzed by ROC curve to determine the accuracy of the ELISAs (area under the curve or AUC, sensitivity, specificity, and the reactivity threshold or cutoff). Further, to determine the Reactivity Index (RI), the cutoff was calculated as: mean absorbance of the 47 CT sera + 2× standard deviation (SD) or 3× SD. Then, RI for each sample was obtained as: sample absorbance ÷ cutoff. Sera were considered reactive when the RI > 1.0 and non-reactive when the RI ≤ 1.0. In this way, to avoid the variation day-to-day of absorbance, when testing all samples (testing phase), RI was calculated for each day of testing using six CT sera (true negatives) tested at the same conditions of the samples to determine the cutoff of the day. Then, the RI was calculated as above (sample absorbance/cutoff).

### Testing phase, agreement between different assays and validation according to gold standard test, and prevalence of the disease

Following the development of the in-house ELISA-S (anti-S-RBD-POLH) and ELISA-N (anti-nucleocapsid) assays, all 400 sera from the epidemiological survey cohort were tested with both assays to compare their performances with LFIA and CLIA. The accuracy of each assay was evaluated, and the level of agreement between them was assessed by Cohen's Kappa coefficient, as described in Statistical Analysis below. Subsequently, a rigorous validation of the assay's accuracy, including determination of positive and negative predictive values (PPV, NPV), was conducted using a subset of samples from individuals with a definitive COVID-19 status confirmed by RT-qPCR (the gold standard; n = 356: 175 RT-qPCR positive (RS) and 181 RT-qPCR-negative (NRS) samples (Supplementary Figure S1A and Supplementary Table S1). Predictive values were also calculated in the context of observed disease prevalence during the epidemiological survey to demonstrate the accuracy in real-world epidemiological conditions (14.3%).

### Evaluation of humoral immune response by ELISA-S and ELISA-N in different periods of pandemic and conditions: primary infection, vaccination, and hybrid immunity

The humoral immune response was characterized across different phases of the pandemic and under various immune conditions using the developed ELISAs. For primary infection, the epidemiological survey cohort with confirmed COVID-19 (n = 175) results were organized based on the disease duration (one to 31 days), and the days post-symptom onset (nine to 35 days). Besides in natural infection, the antibodies were detected after vaccination or hybrid immunity (infection plus vaccine), in sera from 2021–2022. Further, we tested samples from different periods of pandemic (2023–2024), when different omicron subvariants predominate and people were vaccinated and get COVID-19 (vaccine plus breakthrough infection).

### Statistical analysis

The data were analyzed using ROC (Receiver Operating Characteristic) analysis to calculate the sensitivity, specificity, likelihood ratio (LR), area under the curve (AUC), and cutoff of the ELISAs. Results were expressed in absorbance (450-650 nm) and RI. Positive and Negative predictive values (PPV, NPV) were calculated. Agreement between tests was assessed by calculating the percentage of absolute agreement, with 75% considered the minimum acceptable level and > 90% deemed high agreement^24^. The Cohen's Kappa coefficient was used to quantify agreement and interpreted as: < 0.2 is poor agreement; 0.2–0.4, fair agreement; 0.41-0.6, moderate agreement; 0.61-0.8, substantial agreement; > 0.8, great agreement^25^. To compare results between two groups, Mann Whitney's test or Wilcoxon's paired test was used and for more than two groups, Kruskal-Wallis ANOVA followed by the Dunn's *post hoc* test. Analyses were conducted using GraphPad Prism 9 version (San Diego, CA, USA). A p value < 0.05 was considered significant.

## RESULTS

### Characteristics of the individuals whose sera were used for ELISA-S development with the S-RBD-polyhedrin antigen, and serological COVID-19 screening by commercial assays targeting S or N proteins

Supplementary Table S1 summarizes the baseline characteristics of the study participants. In both groups, most individuals were female, representing 56.5% of the uninfected group and 60.0% of the patient group. The age range of uninfected participants was 20–67 years, whereas in the patient group it was 19–77 years. The duration of illness among patients ranged from one to 31 days (median: 10 days). Blood samples were collected between nine and 35 days after symptom onset (median: 18 days). None of the participants showed clinical signs or symptoms of COVID-19 at the time of blood collection. Reported manifestations during illness varied from mild symptoms (fever, headache, rhinorrhea, sore throat, and body pain) to moderate symptoms (memory loss and dyspnea).

As the study focused on the development of serological assays, all sera were initially screened by a LFIA designed to detect anti-SARS-CoV-2 IgM/IgG antibodies. Based on LFIA results, sera were classified as either reactive (RS, patients) or non-reactive (NRS, uninfected controls) (Supplementary Table S1 and Supplementary Figure S1A). A total of 200 sera classified as non-reactive by LFIA (NRS) were further analyzed. Among these, 182 participants (91%) had previously undergone RT-qPCR testing of nasopharyngeal swabs, with 181 confirmed as negative (true negatives). The same 200 samples were subsequently tested by CLIA for detection of IgG antibodies against the N protein. In CLIA, 199 samples were confirmed as non-reactive, whereas one serum (0.5%) tested reactive, corresponding to a participant also confirmed positive by RT-qPCR (1/182 tested). In parallel, sera from 200 individuals reporting COVID-19-related symptoms were classified as reactive by LFIA and designated as patient samples (Supplementary Table S1). Among these, 175 participants tested positive by RT-qPCR, while 26 individuals were not tested. All RT-qPCR-positive patients (175/175; true positives) were reactive in both LFIA and CLIA. Notably, while LFIA detected reactivity in 100% of samples (200/200), CLIA detected reactivity in 95.0% (190/200). Supplementary Figure S1A shows a schematic summary of the testing workflow and results.

### Accuracy of in-house ELISAs developed with S-RBD-POLH antigen (ELISA-S) and with N protein (ELISA-N) in the standardization phase and comparative performance testing with LFIA

**Figure 1:**
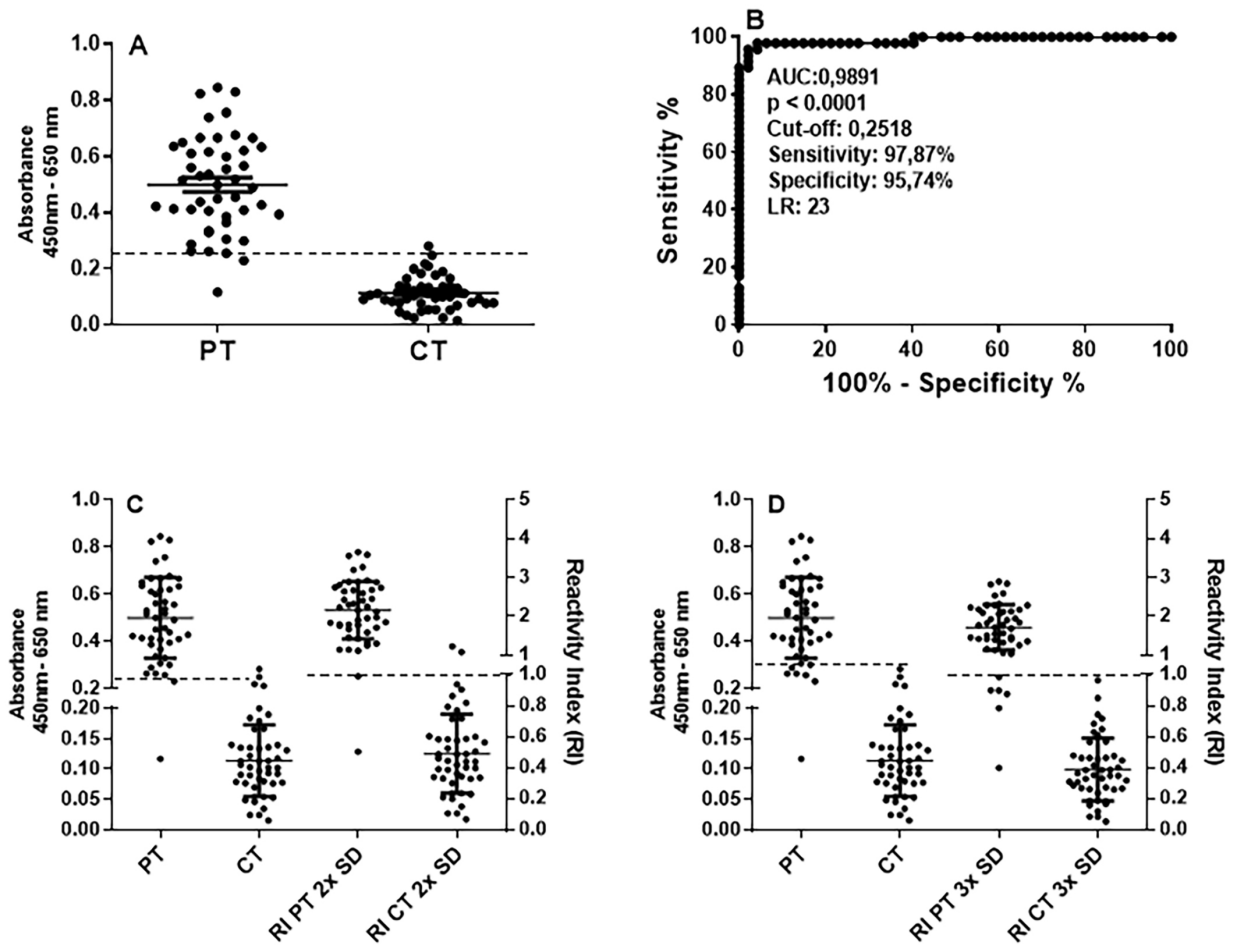
Determination of the accuracy of the enzyme-linked immunosorbent assay-S (ELISA-S) for detection of IgG antibodies to S-RBD-POLH antigen: (A) Absorbance values for sera of the patients (PT; positive by RT-qPCR, n = 47; serum, dilution 1:100), and controls (CT; sera from individuals collected before the COVID-19 pandemics, n = 47); (B) ROC analysis displaying sensitivity, specificity, reactivity threshold (cut off), area under the curve (AUC), and likelihood ratio (LR); (C) separation of patients (PT) and controls (CT) by reactivity index (RI), calculated as the mean of CT + 2× SD; (D) +3× SD. Sera with RI > 1.0 reactive; RI ≤ 1.0 were non-reactive sera. Absorbance values on the left Y-axis, and RI on the right Y-axis. Data are individual values and mean ± SD. SD = standard deviation. Dotted lines indicate the cut-off values.

Supplementary File S1 details all procedures for the development of ELISA-S are, including descriptions and figures (Supplementary Figures S3 and S4). The optimal conditions were determined as purified S-RBD-POLH at 4 μg/mL and serum diluted at 1:100. To assess the diagnostic accuracy of ELISA-S, sera from 47 patients (PT, RT-qPCR positive) and 47 control samples (CT, pre-pandemic samples from 2019) were tested. In addition to confirmation by RT-qPCR, all samples were serologically characterized using a commercial LFIA to verify whether anti-S-RBD antibodies are present. Receiver operating characteristic (ROC) curve analysis was performed to establish the cutoff (reactivity threshold), sensitivity, specificity, likelihood ratio (LR), and area under the curve (AUC). Results demonstrated a clear discrimination between PT and CT sera (Figure 1A), with a cutoff of 0.252. ELISA-S achieved 97.87% sensitivity (95%CI, 88.71%–99.95%), 95.74% specificity (95%CI, 88.46%–99.48%), and an LR of 23 (Figure 1B). The AUC was 0.989 (p < 0.0001), indicating excellent diagnostic accuracy. When the cutoff was alternatively calculated as the mean absorbance of CT sera plus 2 standard deviations (2× SD), sensitivity and specificity were 95.7%, with a cutoff nearly identical to that obtained by ROC (0.2518; Figure 1C). Using a more stringent 3× SD cutoff, sensitivity decreased to 87.0%, but specificity reached 100% (Figure 1D). For subsequent analyses, the 2× SD cutoff was selected, and results were expressed as a reactivity index (RI: Sample absorbance/cutoff).

**Figure 2:**
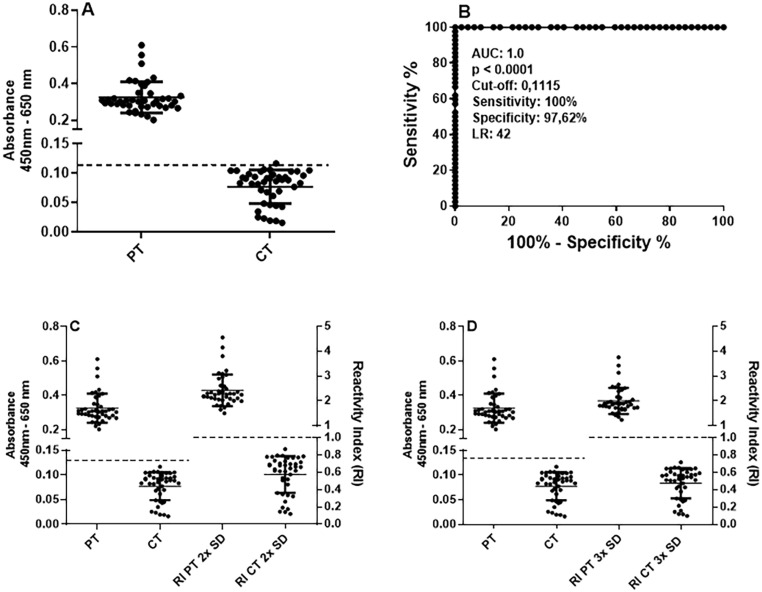
Accuracy of enzyme-linked immunosorbent assay for detecting IgG antibodies to N recombinant protein (ELISA-N): (A) Differential reactivity between sera from 42 patients (PT, RT-qPCR positive), and 42 non-reactive control sera (CT, collected before COVID-19 pandemics; serum dilution 1:50); (B) ROC analysis displaying the area under the curve (AUC), reactivity threshold (cut off), sensitivity, specificity, and positive likelihood ratio (LR); (C) Separation of PT and CT by reactivity index (RI), calculated as the mean absorbance of CT + 2× SD; (D) using +3× SD. Absorbance values are shown on the left Y-axis, and RI on the right Y-axis. Data are individual values and mean ± SD. Dotted lines indicate the cut-off values.

Supplementary File S1 and Supplementary Figure S5 provide assay development procedures for ELISA-N. The same sera used for ELISA-S were analyzed. Figure 2A shows that PT sera (n = 42) and CT sera (n = 42) had distinct reactivity patterns. ROC analysis revealed a reactivity threshold of 0.11, with 100% sensitivity, 97.62% specificity, an LR of 42, and an AUC of 1.0 (p < 0.0001), indicating perfect diagnostic accuracy (Figure 2B). As with ELISA-S, RI was calculated using CT mean absorbance plus 2× SD or 3× SD. The cutoff was 0.13 (2× SD) or 0.16 (3× SD), with 100% sensitivity and specificity in both cases (Figure 2D). For the testing phase, the 3× SD cutoff was adopted. Supplementary Figure S6 shows the results obtained with the 2× SD cutoff. The use of calculated cut-offs with NRS each day of testing avoids interference of absorbance variation day-to-day.

**Figure 3:**
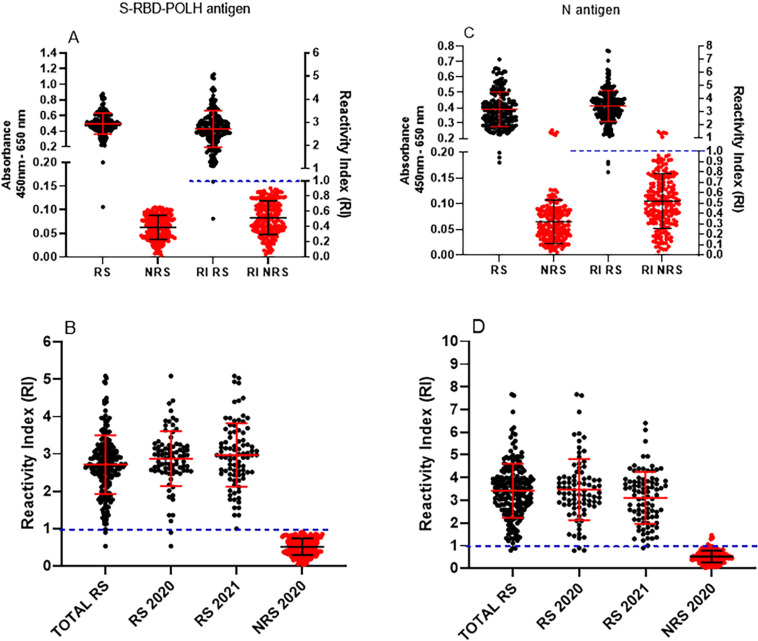
Testing phase of sera by enzyme-linked immunosorbent assay-S (ELISA-S) and immunosorbent assay-N (ELISA-N): Detection of IgG anti-S-RBD and anti-N IgG in serum samples classified as reactive (RS) or non-reactive (NRS) based on lateral flow immunoassay (LFIA) results. Sera from 400 participants (200 patients 200 controls) were tested using LFIA, and classified as reactive sera (RS, 100 from 2020 and 100 from 2021) or non-reactive sera (NRS, 200 from 2020): (A) and (B) ELISA-S results are shown for all RS (n = 200) and NRS (n = 200); (C) and (D) ELISA-N results are shown for all RS (n = 200) and NRS (n = 200); (B) ELISA-S and (D) ELISA-N results are stratified by year-sample collection. Absorbance values are plotted on the left Y-axis, and reactivity index (RI) on the right Y axis. The RI was calculated each day using six negative control sera, with the cut off defined as 2× SD (for ELISA-S) or 3× SD (ELISA-N) above the mean absorbance of non-reactive controls. Data are presented as individual values with means ± SD. The dotted line at RI = 1 represents the threshold distinguishing RS from NRS. Reactive sera (RS, black lines) and non-reactive sera (NRS, red lines).

Under optimized conditions, all 400 samples were tested with ELISA-S to compare performance with LFIA. Among the 200 reactive sera identified by LFIA, two samples (0.5%; 2/200) were non-reactive by ELISA-S. Conversely, all 200 non-reactive sera by LFIA were also non-reactive by ELISA-S (Figure 3A), resulting in 99.5% concordance between the two assays. Antibody levels did not differ significantly between samples collected in 2020 and 2021 (Figure 3B). Similarly, all 400 samples were tested by ELISA-N for detection of anti-N IgG. In this case, five samples reactive by ELISA-N were classified as non-reactive by LFIA (5/200), and three non-reactive by ELISA-N were reactive in LFIA (3/200) (Figure 3C). The overall concordance between ELISA-N and LFIA was 98.0%. Antibody levels were consistent across samples collected in 2020 and 2021 (Figure 3D).

### High accuracy of in-house ELISA-S, in-house ELISA-N, LFIA, and CLIA considering only samples tested for virus presence: validation of in-house ELISAs by commercial assays

During the testing phase, we demonstrated that the in-house ELISAs could detect IgG antibodies in sera from individuals participating in the epidemiological survey (n = 400), showing strong agreement with LFIA results (Figure 3). To further validate these findings, we evaluated and compared the diagnostic performance of all assays considering only participants who were tested for the presence of SARS-CoV-2, using RT-qPCR as the gold standard. Among the sample donors, 356 individuals underwent RT-qPCR testing, which classified them into 181 true negatives and 175 true positives (Supplementary Figure S1A). All RT-qPCR-negative individuals were also classified as non-reactive by LFIA, CLIA, and ELISA-S, resulting in 100% specificity for these assays. Of the 175 RT-qPCR-positive cases, 174 sera were reactive by LFIA (sensitivity: 99.43%), and 169 were reactive by CLIA (sensitivity: 96.57%). ELISA-S and ELISA-N demonstrated sensitivities of 98.86% and 98.29%, respectively, with ELISA-N also showing specificity of 98.34% (Table 1).

**Table 1:** Evaluation of tests for accuracy and agreement

Assay	Standardization phase ROC analyses (ELISAs)	Testing phase percent agreement^f^ Cohen's kappa coefficient	Testing phase Results according to the gold standard reaction^g^	Results considering the prevalence of COVID-19 2020-2021 Goiânia^j^
LFIA S Protein	Sensitivity: 86.43%^a^ [82.51%–89.58%] Specificity 99.57%^a^ [97.63%–99.92%]	LFIA vs. CLIA 97.0% kappa: 0.94 LFIA vs. ELISA-S 99.5% kappa: 0.99 LFIA vs. ELISA-N 98.0% kappa: 0.98	Sensitivity 99.43% Specificity 100% Accuracy: 99.0% PPV: 100%^h^ NPV: 99.0%^i^ LR	Sensitivity 99.43 [96.88%–99.99%] Specificity 99.45% [96.98%–99.99%] Accuracy: 99.44% [98.00%–99.93%] PPV: 99.43 [96.12–99.92] PNV: 99.45 [96.25–99.92] Negative LR: 0.01
CLIA N Protein	Sensitivity 96.70%^a^ Specificity 98.90%	CLIA vs. ELISA-S 97.0% kappa: 0.94 CLIA vs. ELISA-N 96.0% kappa: 0.92	Sensitivity 96.57% Specificity 100% Accuracy: 98.0% PPV: 100% NPV: 96.0% LR	Sensitivity 95.63% [91.57%–98.09%] Specificity 100% [97.98%–100%] Accuracy: 97.08% [95.72%–99.05%] PPV: 100% [97.10%–100%] PNV: 95.77% [91.99%–97.80%] Negative LR: 0.04
ELISA-S S Protein	Sensitivity 97.87% [88.71–99.95%]^b^ Specificity 95.74% [88.46%–99.48%]^b^ LR: 23^c^ AUC 0.9891^d^	ELISA-S vs. ELISA-N 98.2% kappa: 0.98	Sensitivity 98.86% Specificity 100% Accuracy: 99.27% PPV: 100% NPV: 98.04% Negative LR: 0.01	Sensitivity 98.86 [95.93%–99.86% Specificity 100% [97.98%–100%] Accuracy: 99.84% [98.65–100%] PPV: 100% [97.89%–100%] NPV: 99.81% [99.25%–99.95%] Negative LR: 0.01
ELISA-N N Protein	Sensitivity 100% [91.59%–100%]^e^ Specificity 97.62% [87.43%–99.94%]^e^ LR: 42 AUC: 1.0		Sensitivity 98.29% Specificity 98.34% Accuracy: 98.31% PPV: 98.29% NPV: 98.34% LR: 59	Sensitivity 98.29% [95.07%–99.65%] Specificity 98.34% [95.23%–99.66%] Accuracy: 98.33% [96.39–99.39%] PPV: 100% [97.89–100%] NPV: 99.81% [99.25%–99.95%] Negative LR: 0.02 LR: 59.3

a Manufacturer data;

b n = 94 (47 positive and 47 negative controls) [95% IC];

c LR = Positive likelihood ratio;

d AUC = area under the curve;

e n = 84 (42 positive and 42 negative controls);

f n = 200 reactive and 200 non-reactive sera (according LFIA assay);

g,j n = 356 sera (positive or negative by gold standard RT-qPCR);

h Positive Preditive Value;

i Negative Preditive Value;

j Prevalence = 14.3%.

Table 1 shows that the diagnostic accuracy of all assays ranged between 98% and 99%. Importantly, all assays had excellent PPV, NPV, and LR (values detailed in the rightmost column of Table 1). Table 1 also summarizes the results obtained during the ELISA standardization phase (ROC analyses), the degree of agreement between assays in the testing phase (including the kappa coefficient), and the final comparative results using RT-qPCR as the reference method (gray column at left in Table 1). Additionally, for real-world meaning of assay accuracy, we assessed the prevalence of COVID-19 in Goiania city during the serum collection period (2020–2021) and recalculated the diagnostic parameters under those epidemiological conditions (last gray column at right in Table 1). Taken together, in all evaluated scenarios, the performances of the in-house ELISA-S and ELISA-N assays were consistently high, comparable to, and validated by, commercial assays.

### Humoral immune responses in natural infection in 2020–2021 epidemiological survey cohort as well as in hybrid immunity in 2021–2022 and in 2023–2024 cohorts

The humoral immune response requires time to develop following infection, and antibodies may be induced by different viral antigens and at distinct levels during the disease. Both in-house ELISA-S and ELISA-N showed high sensitivity and specificity in detecting antibodies in sera from individuals in the 2020–2021 epidemiological cohort. Notably, no correlation was observed between IgG antibody levels measured by ELISA-S and ELISA-N (Supplementary Figure S7A), indicating that they were detecting distinct antibody profiles. In fact, when analyzed separately, IgG levels were higher in ELISA-N compared with ELISA-S (Supplementary Figure S7B).

**Figure 4:**
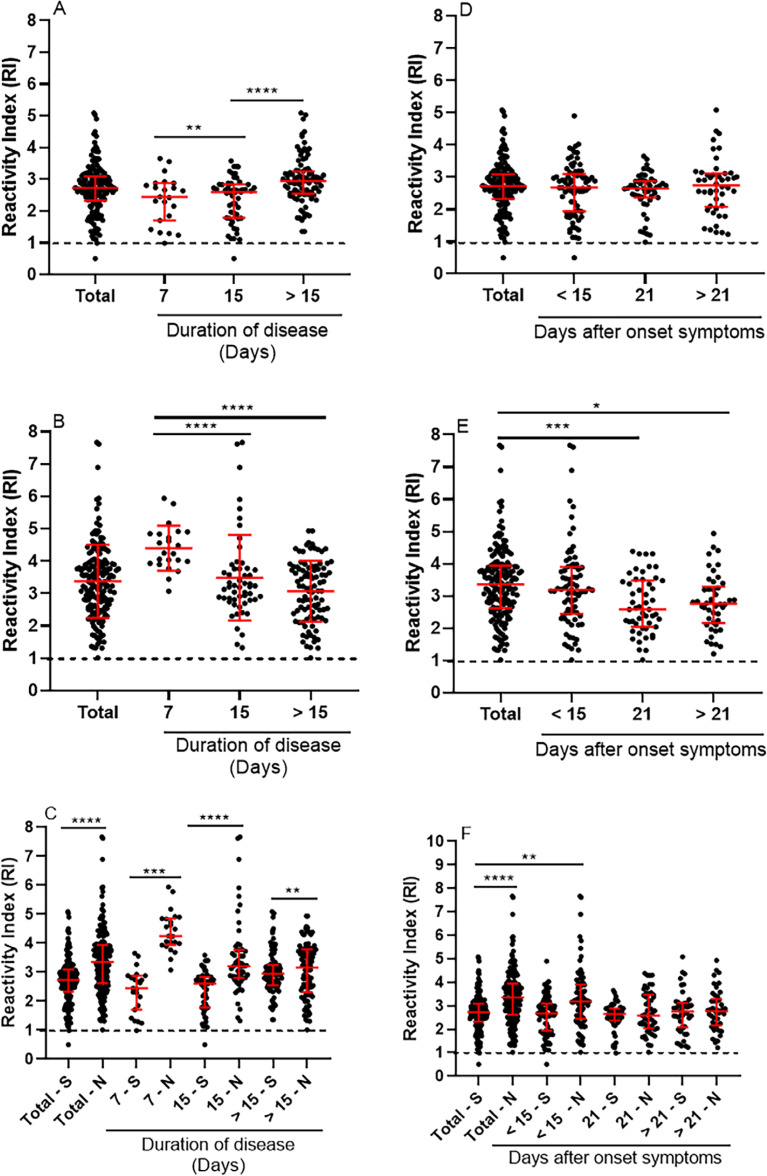
Detection of Anti-S-RBD and Anti-N IgG antibodies according to duration of disease (days with symptoms) and days since symptom onset. Reactivity index (RI) of IgG antibodies from 175 patients who tested positive by RT-qPCR and tested by ELISA. Duration of disease: (A), ELISA-S IgG levels semi-quantified by RI; significant differences between ELISA-S and ELISA-N are shown for each period. **p < 0.01 (≤ 7 days vs. 8–15 days); ****p < 0.0001 (≤ 15 days vs. > 15 days); One non-reactive serum R ≤ 1 (8–15 days); (B) ELISA-N IgG levels. ****p < 0.0001 (≤ 7 days vs. 8–15 days); (C) comparison of IgG anti-S-RBD vs. anti-N. ****p < 0.0001 ((Total N vs. Total S); ****p < 0.0001 (≤ 7 days S vs. N); ****p < 0.0001 (8–15 days S vs. N); **p < 0.0001 (> 15 days S vs. N). Days post-symptom onset: (D) ELISA-S IgG levels semi-quantified by RI; Two non-reactive serum R ≤ 1 (≤ 15 days, 16 −21 days, > 21 days after onset of symptoms); (E) ELISA-N IgG levels, ***p < 0.001 (Total N vs. 21 days after onset symptoms). Two non-reactive serum (< 15 days and 16–21 days after onset of symptoms, R ≤ 1). *p < 0.1 (Total N vs. >21 days); (F) comparison between IgG anti-S vs. anti-N according to days after onset of symptoms; **** p< 0.0001 (Total S vs. Total N). Anova Kruskal-Wallis followed by Dunn's *post hoc* test. S = ELISA-S to IgG anti-S-RBD protein; N = ELISA-N to IgG anti-N protein; vs = versus; Data are shown as individual values, medians, and interquartile ranges. Duration of disease: < 7 days n = 23; 8–15 days n = 56; > 15 days n = 96; Days after onset of symptoms: < 15 days n = 78; 16 – 21 days n = 51; > 21 days n = 46.

To better characterize the humoral immune response to different SARS-CoV-2 antigens, we examined IgG levels in relation to days of symptoms (one to 31 days). Anti-S-RBD-POLH IgG levels increased with symptom duration, whereas anti-N IgG levels decreased (Figures 4A, 4B). Despite this, anti-N IgG levels remained higher than anti-S-RBD-POLH IgG across all groups, with the greatest differences observed during the first and second weeks of symptoms (Figure 4C). Consistent with these findings, anti-S-RBD-POLH IgG levels correlated positively, while anti-N IgG correlated negatively with days of symptoms (Supplementary Figure S8).

Blood samples were collected between nine and 35 days post-symptom onset, when all patients had fully recovered from COVID-19. IgG antibodies against both antigens were detectable from the second week post-symptom onset (Figures 4D, 4E). Anti-S-RBD-POLH IgG levels showed no significant variation over time (Figure 4D). In contrast, anti-N IgG levels declined between the second and third weeks and stabilized thereafter (Figure 4E). Although anti-N IgG levels were consistently higher than anti-S-RBD-POLH IgG, no statistically significant differences were observed between groups stratified by days post-symptom onset (Figure 4F). Furthermore, only anti-S-RBD-POLH IgG, but not anti-N IgG, showed weak positive correlation with days post-symptom onset (Supplementary Figure S8).

Given the observed temporal differences in antibody responses, ROC analyses were performed to assess ELISA performance in three time intervals: ≤ 15 days; 16–21 days; and > 21 days. For ELISA-S, the AUC ranged from 0.99 to 1.0 (Supplementary Figures S9A–D), whereas for ELISA-N the AUC was 1.0 across all groups (Supplementary Figures S9E–F), indicating near-perfect or perfect accuracy for IgG detection over time.

**Figure 5:**
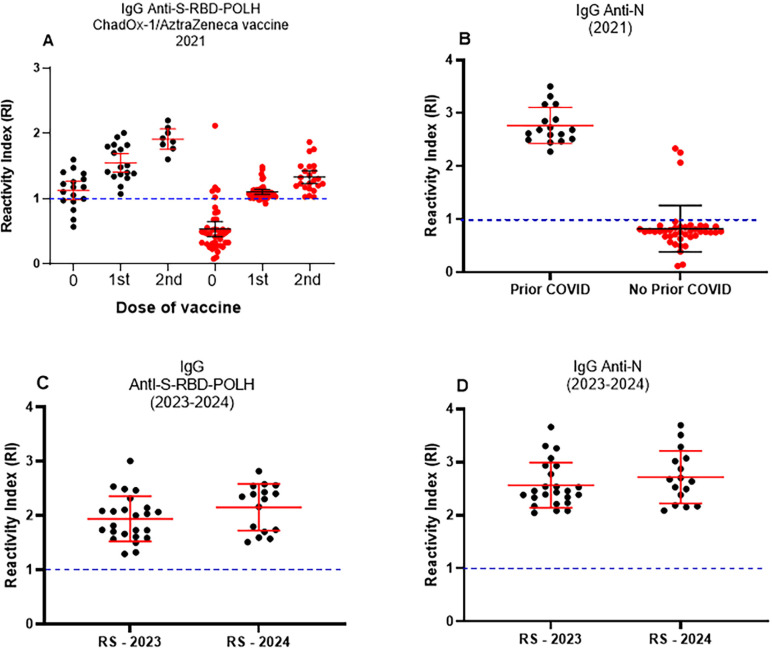
ELISA to SARS-CoV-2 S-RBD-POLH detects IgG antibodies arising against different COVID-19 waves/SARS-CoV-2 variants in hybrid immunity in 2021 and 2023-2024 cohorts: (A) Anti-S-RBD-POLH IgG levels in individuals without (n = 41) or with prior COVID-19 (n = 17) vaccinated with ChAdOx1 nCoV-19. Serum samples were collected at baseline (pre-vaccination, 0), one month after the first dose (n = 17 for prior infection; n = 41 for no prior infection), and one month after the second dose (n = 8 for prior infection; n = 23 for no prior infection); (B) Anti-N IgG levels in the same groups (n = 41 without prior infection; n = 17 with prior infection), indicating natural infection before vaccination (hybrid immunity = natural infection + vaccination). Overall, 147 serum samples were evaluated; (C) Anti-S-RBD-POLH IgG response in individuals with hybrid immunity (n = 40) assessed in 2023–2024; individuals were vaccinated with different types of vaccine and then had COVID-19 (vaccination + infection); (D) Anti-N IgG levels in the same group (n = 40), indicating natural infection. Sera with a reactivity index (RI) > 1.0 were considered reactive; RI ≤ 1.0 were classified as non-reactive. Data are shown as individual values, medians, and interquatis ranges; (A) and (B): black is for prior COVID-19, and red is for without prior COVID-19.

As the pandemic evolved, successive waves of COVID-19 were driven by new SARS-CoV-2 variants of concern (VOCs), while vaccination campaigns progressed. Therefore, it was critical to determine whether ELISA-S could reliably detect IgG antibodies against SARS-CoV-2 strains other than the one used for S-RBD-POLH antigen preparation. Moreover, hybrid immunity—arising from different sequences of infection and vaccination—became increasingly common. For the scenario of infection followed by vaccination, we analyzed a cohort of 58 military police officers (Goias State, Brazil), consisting of 45 males (77.6%) and 13 females (22.4%), from 35 to 41 years (Supplementary Table S1). As shown in Figure 5A, ChAdOx1 nCoV-19/AstraZeneca vaccination induced robust IgG responses detectable by ELISA-S in both previously infected and infection-naïve individuals. In participants with prior COVID-19, a single vaccine dose substantially boosted antibody levels, whereas infection-naïve individuals required two doses to reach comparable levels. Interestingly, some participants without self-reported prior COVID-19 showed anti-S-RBD IgG along with anti-N antibodies, suggesting undiagnosed asymptomatic infections (Figure 5B).

Additionally, sera from 40 individuals collected between 2023 and 2024 were tested by ELISA-S. This group consisted mostly of females (62.5%), from 22 to 45 years (Supplementary Table S1). Participants had been vaccinated with different COVID-19 vaccines available (CoronaVac, AstraZeneca, Janssen, and Pfizer). ELISA-S successfully detected IgG antibodies against S-RBD-POLH in all sera tested (Figure 5C). Moreover, these sera were also reactive in ELISA-N, confirming hybrid immunity (vaccination plus infection; Figure 5D).

Taken together, these results demonstrate that in diverse epidemiological contexts and under varying immunity conditions, in-house ELISA-S and ELISA-N reliably detected IgG antibodies to SARS-CoV-2.

## DISCUSSION

We developed two in-house ELISAs to detect IgG antibodies against the S-RBD and N proteins of SARS-CoV-2 with high accuracy. We established a platform using a stable and high-yield SARS-CoV-2 S-RBD-POLH antigen for robust COVID-19 serodiagnosis and evaluation of humoral immune responses. The sensitivity and specificity of the in-house ELISA-S and ELISA-N exceeded the requirements recommended by the WHO^2^. Comparison of these ELISA performances with LFIA and CLIA, widely used in Brazil and globally, demonstrated strong concordance among assays. The efficacy of our ELISAs was confirmed in sera from participants with RT-qPCR-confirmed COVID-19 and detectable antibodies. In this epidemiological survey cohort, anti-S-RBD and anti-N IgG antibodies were detected early after symptom onset, with longer disease duration correlating with higher anti-S-RBD and lower anti-N antibody levels. The ELISA-S successfully detected IgG antibodies against S-RBD across different COVID-19 waves and vaccination periods (2020–2024).

The use of S-RBD-POLH produced in insect cells and encapsulated within cypovirus OBs offers several advantages for diagnostics. Encapsulation enhances protein stability, preventing degradation and preserving structural integrity over extended periods, which is advantageous for long-term storage and resource-limited settings. Additionally, OB encapsulation simulates a natural crystalline environment, maintaining protein functionality and improving immunoassay sensitivity and specificity. Notably, the Polh component did not show reactivity with antibodies or reagents used.

Antigen and serum concentrations were based on previous ELISA assays detecting S-RBD and N antibodies^21,23,26–28^. ELISA-S showed 98.9% sensitivity and 100% specificity, surpassing commercial ELISAs approved in Brazil, whose sensitivity ranged from 60.2% to 76.8%^6,11^. Vedova-Costa *et al*.^10^ evaluated 38 Brazilian-approved ELISAs, reporting 55%–100% sensitivity and 85%–100% specificity. The antigens were not specified for these commercial assays. In-house ELISAs using S-RBD antigen in Brazil, reported by Moura *et al*.^26^ and Pathirana *et al*.^27^, demonstrated 94.3% and 87% sensitivity, and 100% and 99% specificity, respectively. Oliveira *et al*.^15^ reported 91% sensitivity and 99.25% specificity. Discrepancies were primarily observed in sensitivity, potentially due to variations in recombinant protein expression systems (baculovirus) using the Wuhan-Hu-1 genome^14^. We have previously applied this technology to produce N protein^22^ and used it successfully in an in-house ELISA^29–31^.

Sera from our epidemiological cohort were collected during June–July 2020 and January–April 2021. During these months of 2020, SARS-CoV-2 B.1.1.28 and B.1.1.33 variants were prevalent in Brazil^32^. Genome sequencing increased in March 2021, when alpha (B.1.1.7) and gamma (B.1.1.28.1/P.1) variants predominated^18,19,31,33^. S-RBD fragment used here contained the B.1.1.33 sequence with the D614G mutation, altering the interface of S1 and S2 subunits and affecting RBD conformation and viral infectivity^21,23,26^. The D614G mutation is in alpha, beta, and gamma variants. The N-terminal 30 amino acids corresponded to Polh, providing three-dimensional structural stability^16,20,30^. Recombinant expression in different systems (bacteria, insect, mammalian cells) can impact protein structure and antibody binding, affecting assay sensitivity. Our data indicated comparable antibody levels in 2020 and 2021, suggesting that circulating variant changes did not significantly affect ELISA-S sensitivity. ELISA-S demonstrated similar or superior accuracy compared to assays based on the Wuhan-Hu-1 sequence (released on January 2020, before D614G mutation)^12,13,15^, supporting S-RBD-POLH as a suitable antigen for IgG detection^22,23,26,29,30^.

ELISA-N showed a higher sensitivity and specificity than ELISA-S. Similar in-house N ELISAs reported 90%–99% sensitivity and 92%–98% specificity^12,27,29,33^. In-house ELISAs with N protein worldwide showed good to excellent performances, often outperforming commercial assays^27,29,30^. Accuracy in studies was generally determined using sera collected 10–21 days post-symptom onset. In fact, for ELISA-S the accuracy was perfect after 15 days whereas for ELISA-N, with less than seven days. Between 7–15 days all parameters were perfect for ELISA-N (AUC 1.0; sensitivity and specificity 100%). Meta-analysis by Zheng *et al*.^31^ confirmed that in-house ELISAs with N protein outperformed commercial kits and ELISA with S protein, indicating ELISA-N is appropriate for early IgG detection post-infection.

Sera were tested with the LFIA Wondfo SARS-CoV-2 antibody test, which is widely used in Brazil. Two large seroprevalence surveys (> 25,000 participants) using LFIA estimated sensitivity at 84.8% and specificity at 99.0%^25^. In our study, LFIA accuracy was 86.4% sensitivity and 99.6% specificity. This high LFIA sensitivity likely resulted from serum use rather than capillary blood recommended by the manufacturer. We have used sera for comparison of the serological tests. Moreover, 356 participants were positive for viral presence, LFIA achieved 99.43% sensitivity and 100% specificity, validating its use as a screening tool. Percentage agreement between LFIA and ELISA-S or ELISA-N was 99.5% and 98.0%, respectively. LFIA detects IgG and IgM, whereas ELISA-S detects only anti-S-RBD IgG, yet ELISA-S demonstrated high sensitivity. CLIA and ELISA-N both detect IgG anti-N, the kappa coefficient was 0.94, indicating strong concordance. There are several reports comparing different immunoassays with in-house ELISAs. In general, kappa coefficients have varied from satisfactory to almost perfect agreement between LFIA, CLIA, and in-house ELISAs. However, the percentage concordance or kappa coefficients were low in some comparisons. Variability in concordance among immunoassays is influenced by the use of commercial kits, antigen targets, sample timing, disease severity, gold standards, population differences, and serum interferences^22,27,29,30,33^. Using two ELISAs for different targets as S-RBD-POLH and N proteins enhances result confirmation, especially for borderline cases and low prevalence of the disease. The in-house ELISAs showed high accuracy and PPV and NPV, considering or not the disease prevalence in 2020-2021. Although it can be considered that we have evaluated a low number of samples during standardization phase (47 negative and 47 positive sera) the method was validated by the large epidemiological cohort assessed by our ELISAs and commercial assays (400 samples).

Levels of anti-SARS-CoV-2 IgG antibodies (to S1, RBD, N) correlate with neutralizing antibody levels, with moderate to high agreement (kappa > 0.6)^23,26,34,35^. Then, ELISA-S/N can estimate immune status. In epidemiological survey patients with mild/moderate COVID-19 symptoms (> 15 days in 54.86% of patients) showed early detection of anti-S-RBD and anti-N IgG. Anti-N IgG levels were higher than anti-S-RBD IgG during the first week and decreased with prolonged disease, whereas anti-S-RBD IgG increased with symptom duration. Moreover, there was a positive correlation between the levels of IgG anti-S-RBD and the days of symptoms. In opposite, a negative correlation was detected between anti-N IgG levels and duration of the disease. Few studies have evaluated the relationship between duration of symptoms and antibody production. Our results agree with previous studies associating disease duration and IgG antibodies to SARS-CoV-2, and persistence of antibodies in patients with COVID-19^28–33^. Jain *et al*.^34^ evaluated more than thousand blood donors’ convalescents of COVID-19 and confirmed the positive association between levels of IgG anti-S antibodies and duration of disease. In opposite, it has been shown that levels of anti-RBD IgG antibodies do not correlate with duration of the disease^35^. Our data corroborate the findings of association between disease duration and antibody production and suggest that ELISA-N could be used for early detection of antibodies after the beginning of the COVID-19 symptoms.

Concerning the time after onset of COVID-19 symptoms, when blood was collected, this study showed that the levels of anti-S-RBD IgG remained stable during the first, second, and ≥ third week (nine to 35 days). Nevertheless, the levels of IgG antibodies to N protein were higher than those to S-RBD antigen at the second week post-symptoms onset, and then slightly decreased. Notably, a positive correlation between levels of IgG anti-S-RBD and days after symptom onset was detected, but not for the IgG anti-N levels. Anti-SARS-CoV-2 or neutralizing IgG antibody production is quite heterogeneous, but these antibodies are mostly detected during the first week of infection, with a peak between the second/third, a plateau, and decrease. In general, the detection rates of IgG anti-N antibodies are earlier than IgG anti-S or anti-S-RBD, but later is the opposite^31^. Although the antibody levels decrease, they can persist up to 12 months^2,11,29^. Associations between levels of anti-SARS-CoV-2 and days post-symptom onset have been detected and can be dependent on the degree of severity of the disease (mild/moderate/severe) or whether patients are asymptomatic^28^. Here, the cases of COVID-19 were mostly mild with highest detection rate during the second week after onset of symptoms (44.6%). As in this study infected individuals show mild/moderate symptoms, fast recover, and low levels of antibodies, this is aligned with the studies showing that asymptomatic, mild/moderate and severe disease differs in the levels and time of antibody persistence^34,35^. Since all participants in the epidemiological survey no longer showed symptoms when donating blood for research, by combining ELISA-S and ELISA-N we could show the antibody profile of patients with mild/moderate COVID-19 evaluated during short time after the end of the disease symptoms.

The efficacy of diagnosis assays and vaccination have been faced to the emergence of VOCs of SARS-CoV-2. ELISA-S detected antibodies following vaccination with ChAdOx-1 nCoV-19 in individuals with or without prior COVID-19. Results were similar to Mazzoni *et al*.^36^ who showed that after infection one dose of vaccine can improve antibody production. Moreover, after breakthrough infection in vaccinated individuals, our assay detected that antibodies probably emerged against the vaccine antigens as well as the SARS-CoV-2 VOCs prevalent in 2023–2024. As ELISA-N detects natural infection, this assay was crucial to confirm the hybrid immunity. Interestingly, the titers of antibodies in sera from 2023-2024 were slightly superior to those in sera from vaccinated individuals with hybrid immunity in 2020–2021, at the same time of circulating VOC that was used for ELISA-S development. In agreement, despite of high variation in antibody levels, Girl *et al*.^21^ have shown that a breakthrough infection after vaccination increased and improved neutralizing antibodies to all VOCs evaluated, including omicron (alpha, beta, delta, and omicron). Similar results were reported recently, showing that a single JN.1 or repeated infections with other omicron variants after vaccination may extend the breadth of neutralizing antibodies against variants of omicron^37^. Together with our data, it has been suggested that hybrid immunity can improve humoral immune response against different SARS-CoV-2 VOCs^37–39^. Moreover, data suggest that diagnostic assays can continue working in the context of hybrid immunity.

This study has limitations in studying humoral immune responses. One limitation is that the clinical data and course of disease were obtained from a self-reported questionnaire survey, not from medical records, which can introduce recall bias and data missing. We could not perform a longitudinal study to better establish the profiles of responses since the study was transversal in the epidemiological survey. Consequently, the days after post-symptoms onset were evaluated for a short time. Nevertheless, these findings suggested a profile of humoral immune response with anti-SARS-CoV-2 IgG antibodies in a sample of patients who mostly had mild symptoms of COVID-19, and fast recovery. In fact, these findings seem to represent the profile of the population in Goiania city, Goias State, Brazil, who mostly had mild COVID-19 (> 95%) during the period of the survey and thereafter. For the cohorts of hybrid immunity, the limitations were the same for the studies cited above, a variety of vaccines, different schedules that could be with homologous or heterologous immunizations, complete or not dose schedules, as well as time before/after vaccination that the breakthrough infection occurred. This was because the material of the cohort was residual diagnostic material kept in the laboratory. We need to emphasize that the cohorts included mild/moderate COVID-19 cases while severe or hospitalized cases were not represented. Individuals who had infection caused exclusively by more recent variants (e.g., Omicron sublineages) are also not included in this study. Thus, the potential of the assays to diagnose these infections remains uncertain and warrants investigation in future studies. All these points can interfere in antibody immune response and diagnosis. Despite these, ELISA-S effectively detected antibodies to S-RBD-POLH after natural infection, vaccination, or hybrid immunity, whereas ELISA-N was crucial to determine natural infection when an inactivated virus vaccine was not used.

## CONCLUSION

In conclusion, we have developed two high-accuracy ELISAs (to S-RBD-POLH and N proteins) enabling early evaluation of IgG antibody profiles in mild COVID-19 as well as hybrid immunity across COVID-19 waves. While RT-qPCR is the diagnostic gold standard, serological assays are crucial for post-infection antibody detection and distinguishing natural infection from vaccination responses^36,38^. The high-accuracy ELISAs should be important in detecting low levels of antibodies after long periods post vaccination, contributing to determine the longevity of anti-S-RBD IgG levels. Most importantly, the use of cypovirus OB provides a unique platform that enables the encapsulation of multiple proteins, enabling the simultaneous detection of various viral antigens or antibodies in a single assay. This flexibility is critical for developing comprehensive diagnostic tools, especially in cases in which simultaneous detection of different responses is needed. The ELISA-S-RBD-POLH can be updated for the detection of different variants of the virus that can be circulating, which opens new avenues to produce immunoassays with multiple antigens. Cost-effectiveness analysis favors ELISA use in Brazilian public health settings^40^. Thus, our study provides a platform for accurate diagnosis, humoral immune response characterization, and hybrid immunity detection in SARS-CoV-2 infection. It is crucial to continue evaluating hybrid immunity in COVID-19 so that the vaccines can be updated and public health system can deliberate on vaccination schedules. Immunoassays such as these developed here are of continued relevance for long-term surveillance, hybrid immunity assessment, and fostering national technological self-reliance.

## Data Availability

The complete anonymized dataset supporting the findings of this study is available from https://doi.org/10.48331/SCIELODATA.OAJP5A
