# Supplementary material for: Occlusion body-incorporated SARS-CoV-2 S-RBD via cypovirus polyhedrin-derived peptide: a novel insect cell-expressed antigen for high-accuracy COVID-19 diagnosis, and humoral immune response and hybrid immunity evaluation
Source: Rev Inst Med Trop Sao Paulo. 2026 Feb 16;68:e12. doi: 10.1590/S1678-9946202668012 (PMC12919303; doi:10.1590/S1678-9946202668012)
Supplement: Supplementary Material 1 [file 1678-9946-rimtsp-68-S1678-9946202668012-Suppl01.pdf]

REVISTA DO INSTITUTO DE MEDICINA TROPICAL DE SÃO PAULO

JOURNAL OF THE SÃO PAULO INSTITUTE OF TROPICAL MEDICINE

SUPPLEMENTARY MATERIAL

https://doi.org/10.48331/SCIELODATA.OAJP5A

# Occlusion body-incorporated SARS-CoV-2 S-RBD via cypovirus polyhedrin-derived peptide: a novel insect cell-expressed antigen for high-accuracy COVID-19 diagnosis, and humoral immune response and hybrid immunity evaluation

**Murilo Barros-Silveira [1*], Fabricio da Silva Morgado [2*], Laurine Lacerda Pigosso[3], Fernanda Cortez Roriz Pontes[2], Ethiane Rozo dos Santos[2], Rosana Pereira Morais[1], Yves Mauro Fernandes Ternes [1], Célia Maria de Almeida Soares [3], Bergmann Morais Ribeiro [2**], Fátima Ribeiro-Dias [1**]**

[1]Universidade Federal de Goiás, Instituto de Patologia Tropical e Saúde Pública, Laboratório de Imunidade Natural, Goiânia, Goiás, Brazil

[2]Universidade de Brasília, Instituto de Ciências Biológicas, Departamento de Biologia Celular, Laboratório de Baculovírus, Distrito Federal, Brazil

[3]Universidade Federal de Goiás, Instituto de Ciências Biológicas II, Goiânia, Goiás, Brazil

*These authors contributed equally to the study

**These are senior authors of the study

**Correspondence to:** Fátima Ribeiro-Dias Universidade Federal de Goiás, Instituto de Patologia Tropical e Saúde Pública, Laboratório de Imunidade Natural, Rua 235, CEP 74605-050, Goiânia, GO, Brazil

**E-mail:** fdias@ufg.br

**Received:** 12 April 2025

**Accepted:** 25 November 2025

**Editor:** Tania Regina Tozetto Mendoza

## Production of recombinant S-RBD protein with poyhedrin

Recombinant protein production began with amplifying the receptor-binding domain of the SARS-CoV-2 S gene via RT-PCR using total RNA from a nasopharyngeal swab sample kindly supplied by the Central Laboratory of Public Health in the Federal District, Brazil in 2020[6], using primers containing *Pst*I and *Sph*I restriction sites at the 5' ends of each primer, generating a fragment of approximately 950 bp. This fragment was cloned into a modified pFastBac™ Dual plasmid (Thermofischer Scientific, Massachusetts, USA), under the control of the Autographa californica multiple nucleopolyhedrovirus (AcMNPV)'s *polh* promoter. This plasmid also contains the *polyhedrin* gene of the *Thyrinteina arnobia* cypovirus 14 (TharCPV-14)[15,17,20] cloned in reverse orientation, under the control of the AcMNPV's *p10* promoter. The insertion of the S gene fragment into the plasmid caused a fusion of the S gene with a copy of the Alpha-helix 1 of *polyhedrin* gene of TharCPV. The recombinant plasmid was used to generate a recombinant AcMNPV baculovirus via Bac-to-Bac technology (Thermofischer Scientific, Massachusetts, USA), following the manufacturer's instructions, producing the BACTharCPVPOLHS-RBD virus. Infection of susceptible insect cells with the recombinant baculovirus resulted in the production of cytoplasmic occlusion bodies (OBs) containing the S-RBD antigen. A second recombinant baculovirus was constructed without a copy of the TharCPV *polyhedrin* gene using the same methodology described above and called BACS-RBD. The sequence of this fragment containing the S-RBD was deposited in GISAID hcov-19/BRAZIL/DFBR-PCT13/2020.

## Insects, infection, and purification of recombinant S-RBD-POLH

Approximately 1,000 *Spodoptera cosmioides* eggs were obtained from pragas.com® (Piracicaba, SP, Brazil). Larvae were maintained at 25 °C under a 12/12 h light/dark cycle and fed an artificial diet[1a]. When the larvae reached the 4th instar, they were infected with the recombinant baculoviruses BACTharCPVPOLHS-RBD and BACS-RBD via injection of 10 µL of medium containing the recombinant virus ($10^6$ PFU) into the hemocoel. Five days post-injection (dpi), the dead insects were homogenized in an equal volume of ddH2O (w/v), filtered through gauze, and centrifuged at 5,000 × *g* for 10 min[2a]. The supernatant was discarded, and the pellet was resuspended in the same volume of 0.5% SDS detergent and centrifuged at 5,000 × *g* for 10 min. These procedures were repeated three times. The resulting



This is an open-access article distributed under the terms of the Creative Commons Attribution License.

pellets (S-RBD-POLH and S-RBD) were resuspended in 0.5 M NaCl, centrifuged again as described above, and resuspended in phosphate-buffered saline [PBS 1X] (NaCl 137 mM, KCl 2.7 mM, Na2HPO4 10 mM, KH2PO4 2 mM, pH 7.4). For purification, the suspension containing the OBs (S-RBD-POLH) was loaded onto a discontinuous sucrose gradient (60%–90% sucrose in PBS 1X) and centrifuged at 96,000 × *g* for 1 h. The band containing the recombinant OBs (S-RBD-POLH) was removed from the gradient, diluted fivefold with ddH2O, and centrifuged at 5,000 × *g* for 10 min. The purified recombinant OBs were analyzed by 12% SDS-polyacrylamide gel electrophoresis (SDS-PAGE) as described[3a] and immunodetected by Western blotting[4a] (Supplementary Figure S2). Total proteins were quantified using the BCA method (Pierce BCA Protein Assay Kit, Thermo Scientific, Rockford, USA) after diluting the OBs in 0.1 M sodium carbonate-bicarbonate buffer, pH 9.6, for 30 min at room temperature. The concentration of the S-RBD protein was determined by referencing a standard curve of bovine serum albumin (BSA, Sigma-Aldrich, Missouri, USA).

## In-house enzyme-linked immunosorbent assay (ELISA-S): assay conditions

To optimize ELISA conditions for detecting IgG antibodies against S-RBD-POLH, we tested various concentrations of the S-RBD-POLH protein (4 µg/mL, 2 µg/mL, and 1 µg/mL) and serum dilutions (1:50, 1:100, 1:200, and 1:400). The best performance, in terms of separating reactive and non-reactive sera, was achieved with 4 µg/mL protein and serum dilutions 1:50 and 1:100. One representative experiment for testing serum dilution is shown in Supplementary Figure S3, in which S-RBD-POLH purified as the antigen prepared for the best conditions of our ELISA. The results showed similar antigenicity between S-RBD proteins without POLH as well as POLH (Supplementary Figure 3). Supplementary Figure S4 is a representative experiment showing that purification was necessary to improve the performance of S-RBD-POLH. In Supplementary Figures S4A-C are shown results using S-RBD-POLH not purified. In Supplementary Figures 4D-F, the purified S-RBD-POLH protein at 4 µg/mL demonstrated the best separation between patient (PT) and control (CT) sera at a 1:100 serum dilution (Supplementary Figures 4D-F), with an absorbance ratio (PT/CT) of 12.14x at 4 µg/milk The optimal conditions were validated by repeating the experiments using a 1:50 serum dilution (as in Supplementary Figure S3), in which the absorbance ratio was 9.23× (Supplementary Figures S4G-I). These conditions (S-RBD-POLH purified at 4 µg/mL and serum 1:100) were adopted for evaluating the accuracy of ELISA-S in the standardization phase.

## In-house enzyme-linked immunosorbent assay with N protein (ELISA-N): assay conditions

For ELISA-N, the N protein concentrations tested were 5 µg/mL, 3 µg/mL and 2 µg/mL, with serum dilutions of 1:25, 1:50, 1:100, and 1:200. The best performance was observed at 3 µg/mL with serum dilution of 1:50, yielding good separation between PT and CT sera (Supplementary Figure S5). Although serum at 1:25 (absorbance ratio of PT/CT = 4.59×) dilution also showed a good performance, with 1:50 the absorbance ratio was more than five-fold increased (5.51×). Thus, the antigen concentration was at 3 µg/mL, and the serum dilution at 1:50. Supplementary Figure S6 shows the results using 2× SD, which indicated higher number of RS in the LFIA NRS group than when using 3× SD for cut off (Figure 2A). The results are shown with RI calculated with the cut off as mean absorbance + 3× SD, since there was no difference in sensitivity and specificity in standardization phase with both cut off (2× or 3× SD).

## REFERENCES


1. Greene GL, Leppla NC, Dickerson WA. Velvetbean caterpillar: a rearing procedure and artificial medium. J Econ Entomol. 1976;69:487-8.
2. O'Reilly DR, Miller LK, Luckow VA. Baculovirus expression vectors: a laboratory manual. New York: Oxford University Press; 1994.
3. Laemmli UK. Cleavage of structural proteins during the assembly of the head of bacteriophage T4. Nature. 1970;227:680-5.
4. Kurien BT, Scofield RH. Western blotting. Methods. 2006;38:283-93.

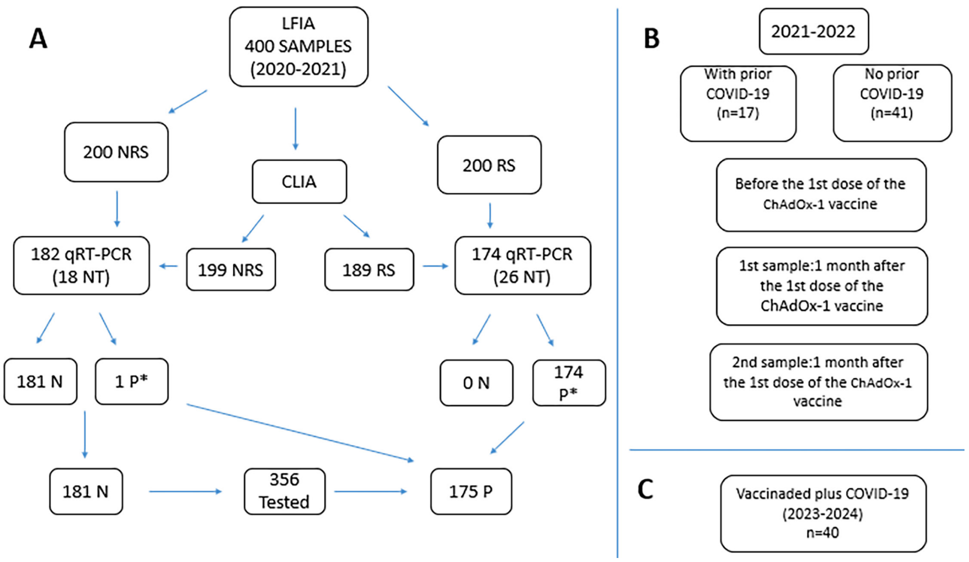


**Supplementary Figure S1** – Cohort of epidemiological survey (testing by LFIA, CLIA, and confirmation by RT-qPCR), cohort of natural infection/hybrid immunity (infection + vaccination), and cohort of hybrid immunity (vaccination + infection), in different periods of pandemic: (A) A total of 400 samples were classified as reactive sera (RS) and non-reactive sera (NRS) by the LFIA. These samples were retested by CLIA and again classified as RS and NRS. Of the 400 samples, 181 were negative (true negatives) across all the tests, and 175 samples were positive (true positives), resulting in 356 samples tested by RT-qPCR. One false negative in LFIA, but *true positive in CLIA, and RT-qPCR (174* positives + 1* positive = 175 true positives). No false positive was detected in LFIA or CLIA; however, CLIA produced six false negatives. LFIA = Lateral flow immunoassay; CLIA = Chemiluminescence imunoassay; qRT-PCR = Quantitative real-time reverse transcription PCR; P = Positive; N = Negative; (B) Cohort of natural infection + vaccination; (C) Cohort of vaccination plus natural infection.

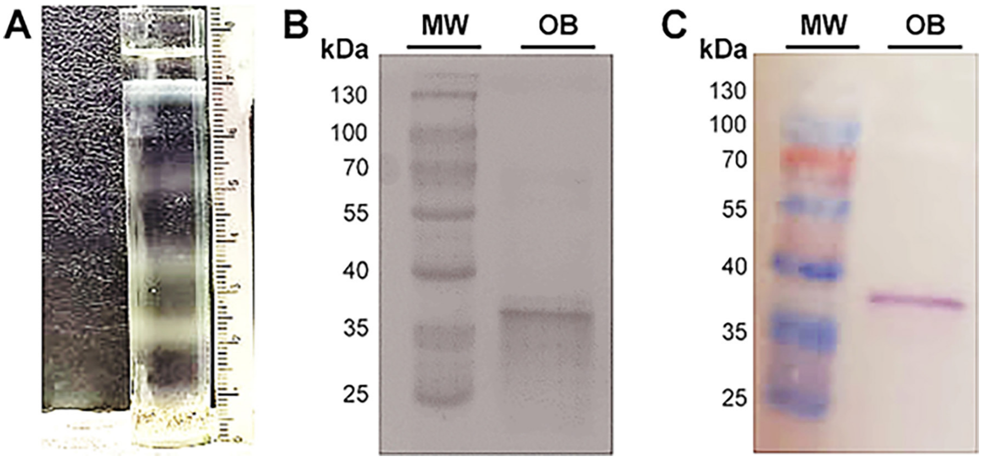


**Supplementary Figure S2 –** Protein analyses of purified BACTharCPVPOLHS-RDB: (A) Purification of occlusion bodies (OB) using a discontinuous sucrose gradient (60% to 90%); (B) SDS-PAGE (12%) analysis of purified OBs, showing a distinct band at approximately 40.06 kDa, corresponding to the S-RBD fusion protein; (C) Western blot of purified OBs, probed with anti-His antibody. The detection of a band at the expected molecular weight confirms successful incorporation of the S-RDB fusion protein into the OBs. MW = molecular weight.

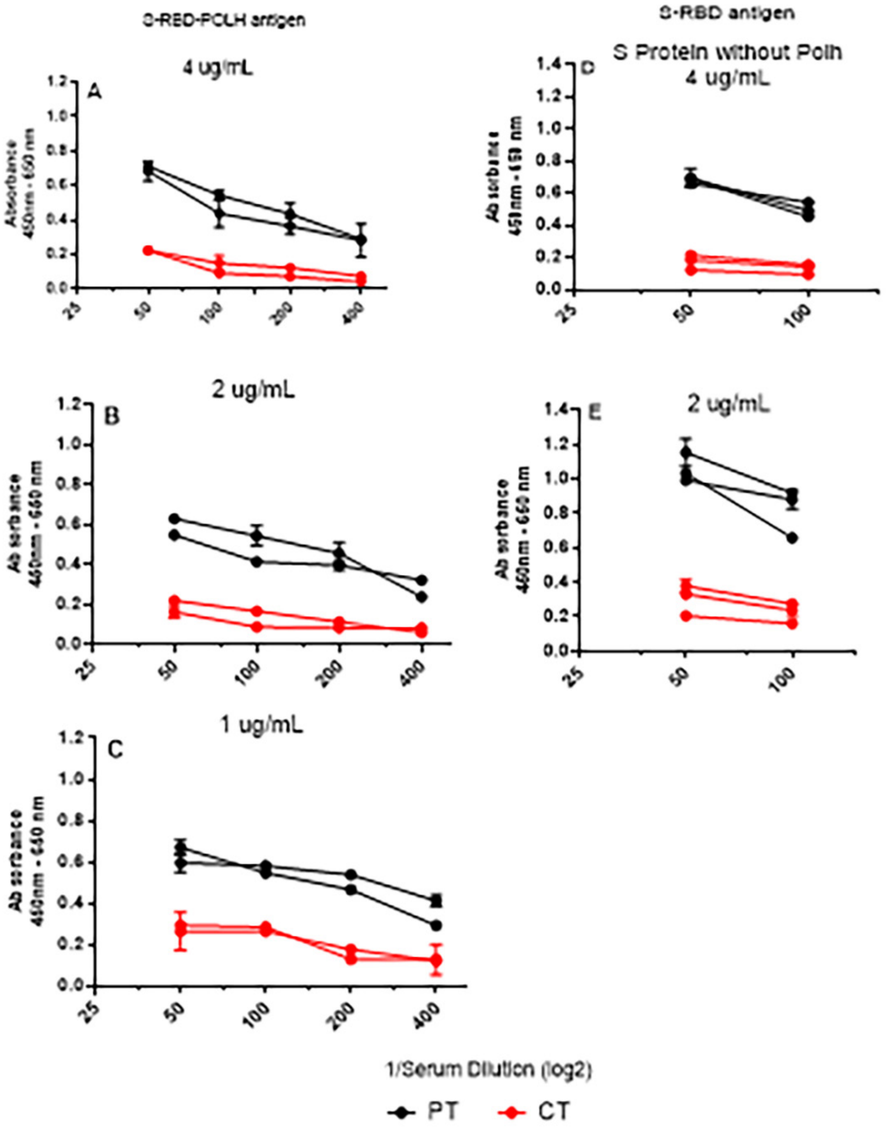


**Supplementary Figure S3** – *In-house* enzyme-linked immunosorbent assay-S (ELISA) for detection of anti-SARS-CoV-2 IgG antibodies against S-RBD-POLH protein: Serum dilution test: (A), (B), and (C) ELISA tests were performed using S-RBD protein with polyhedrin (Polh) purified at four concentrations of 4 µg/mL (A), 2 µg/mL (B), 1 µg/mL (C); (D) and € The antigen without Polh. The reproducibility of the dilution test was assessed with serum of 1:50, 1:100, 1:200, 1:400 serum dilutions. The washing solution consisted of PBS with 0.05% Tween 20. Blocking was performed with 5% powdered milk and 1% BSA in sodium carbonate-sodium bicarbonate buffer, 2 h at room temperature. Sera diluted in PBS with 0.05% Tween 20 and 0.1% BSA for 1 h and incubated at room temperature at dilutions of 1:50, 1:100, 1:200, and 1:400. Anti-IgG-peroxidase conjugate was diluted 1:500n and the reaction was developed with TMB for 10 min. Absorbance readings were taken at 450 nm and 650 nm. Two sera from patients with confirmed SARS-CoV-2 infection by RT-qPCR (Patients, PT) and two sera from healthy individuals collected before the COVID-19 pandemic (Controls, CT, red lines) were tested.

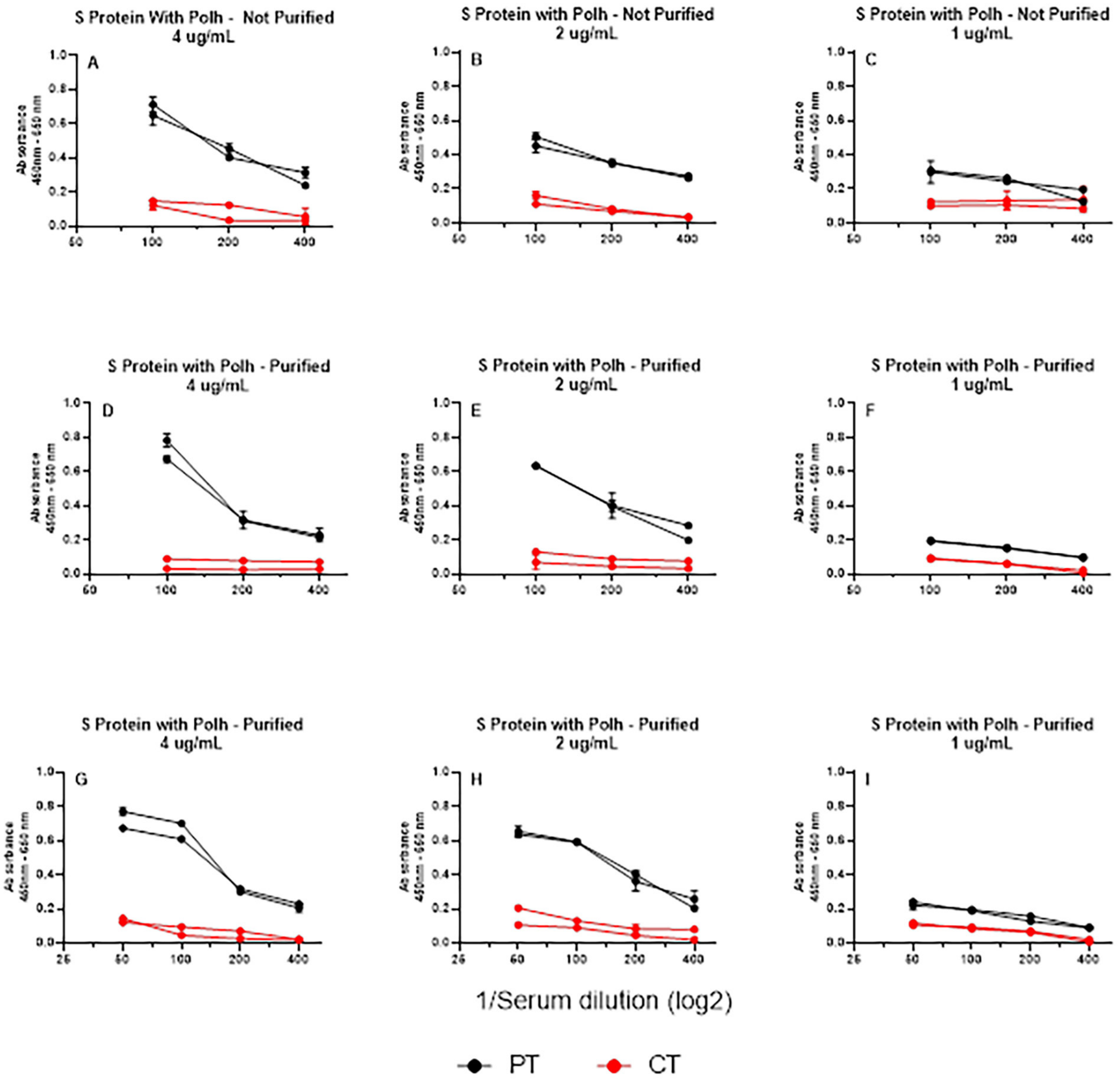


**Supplementary Figure S4** – *In house* enzyme-linked immunosorbent assay-S (ELISA-S) for detection of anti-SARS-CoV-2 IgG antibodies against S-RBD protein. Definition of experimental conditions. S-RBD protein with Polh without purification at 4 µg/mL (A), 2 µg/mL (B), 1 µg/mL (C). S-RBD protein with Polh purified at 4 µg/mL (D), 2 µg/mL (E), 1 µg/mL (F). The reproducibility of the test with the S-RBD protein with Polh purified at 4 µg/mL (G), 2 µg/mL (H), 1 µg/mL (I), adding sera diluted 1:50. The washing solution was PBS with 0.05% Tween 20. Blocking was done with 5% powdered milk and 1% BSA in sodium carbonate-sodium bicarbonate buffer, 2 h, at room temperature. The sera diluted 1:50, 1:100, 1:200, and 1:400 in PBS with 0.05% Tween 20 and 0.1% BSA were incubated for 1 h, at room temperature. The anti-IgG-peroxidase conjugate diluted 1:500, and reaction was developed with TMB 10 min. Absorbance readings at 450 nm and 650 nm. Two sera from patients qRT-PCR positive for SARS-CoV-2 (Patients, PT), and two sera from healthy individuals collected before the COVID-19 pandemic (Controls, CT) were used.

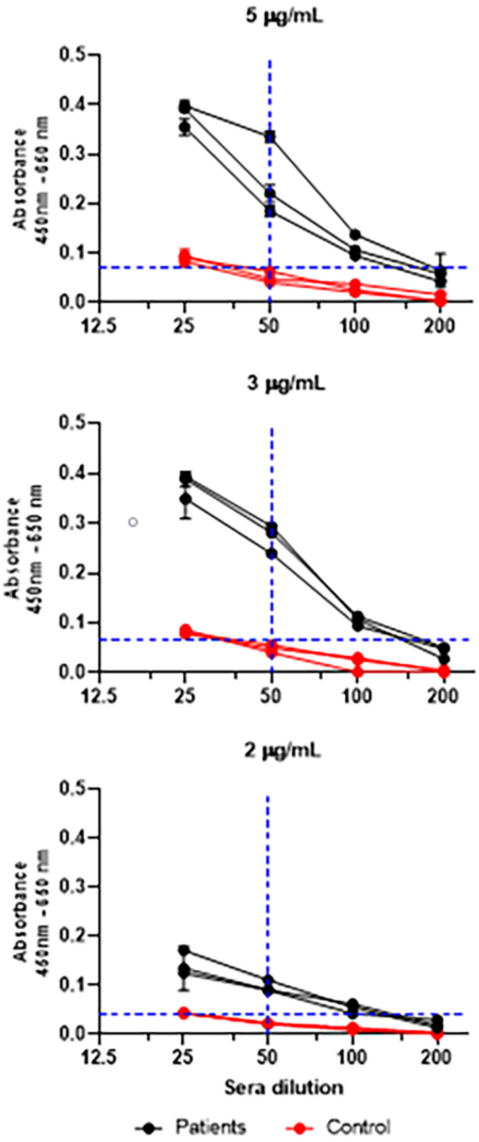


**Supplementary Figure S5** – Immunoenzymatic assay for detection of antibodies against recombinant N protein (ELISA-N): Optimization of antigen concentration and serum dilution. ELISA was performed using N protein at different concentrations: 5 μg/mL (A), 3 μg/mL (B), 2 μg/mL (C). The washing solution used PBS with 0.05% Tween 20. Blocking was conducted with 5% powdered milk and 1% BSA, in sodium carbonate-sodium bicarbonate buffer pH 9.0 for 2 h, at room temperature. Sera were diluted 1:25, 1:50, 1:100, and 1:200 in PBS with 0.05% Tween 20 and 0.1% BSA, and incubated for 1 h, at room temperature. Anti-IgG-peroxidase conjugate was diluted 1:500, and incubated for 1 h, at room temperature. The reaction was developed with TMB for 10 min. The absorbance was measured at 450 nm/650 nm. Three sera from qRT-PCR-positive patients SARS-CoV-2 and two sera collected from healthy individuals (before the COVID-19 pandemic) were tested. The dotted lines (blue) indicate the optimal antigen concentration and serum dilution for the best separation between patients (PT, black lines) and controls (CT, red lines), chosen for the next steps.

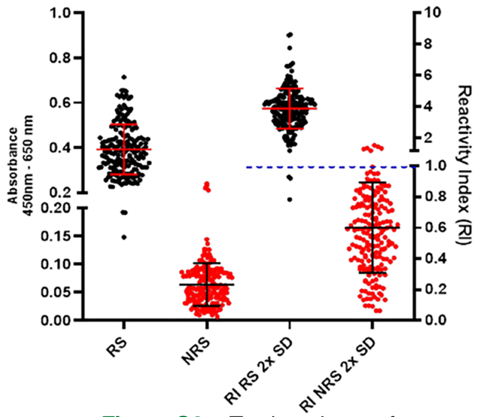


**Supplementary Figure S6** – Testing phase of enzyme-linked immunosorbent assay-N (ELISA-N): Detection of IgG anti-N protein antibodies (using 2× SD as threshold) in serum samples classified as reactive (RS) or non-reactive (NRS) by LFIA. Sera from 400 individuals (200 patients and 200 controls were tested by lateral flow immunoassay (LFIA), and subsequently categorized into reactive sera (RS, 2020: n = 100, 2021: n = 100; in black) and non-reactive sera (NRS, 2020: n = 200, in red). All sera were tested by ELISA-N using 2× SD as threshold. Data are shown as absorbance on the left Y-axis, and reactivity index (RI) on the right Y-axis. The RI was calculated each day using the mean absorbance of six non-reactive control sera+ 2× SD. Data are shown as individual values with means ± SD. The dotted line (in blue) indicates RI = 1, serving as the cutoff point between RS and SNR in ELISA-N.

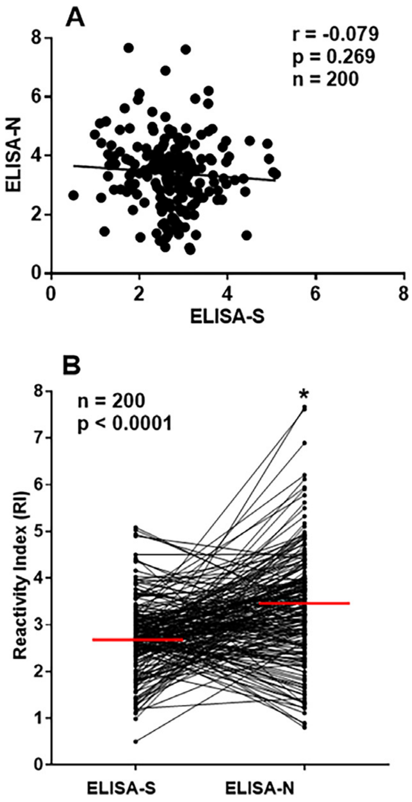


**Supplementary Figure S7** – Comparison of serum reactivity levels detected by enzyme-linked immunosorbent assay S (ELISA-S) and enzyme-linked immunosorbent assay N (ELISA-N): (A) Correlation between IgG anti-S-RBD and anti-N protein levels (reactivity index, RI semi-quantitative IgG detection); n = 200 reactive sera, Spearman test; (B) Reactivity index (RI) values for each reactive sample in both ELISA-S and ELISA-N tests; n = 200, *p < 0.0001. Wilcoxon test. Data are shown as individual measurement (black) and medians (red).

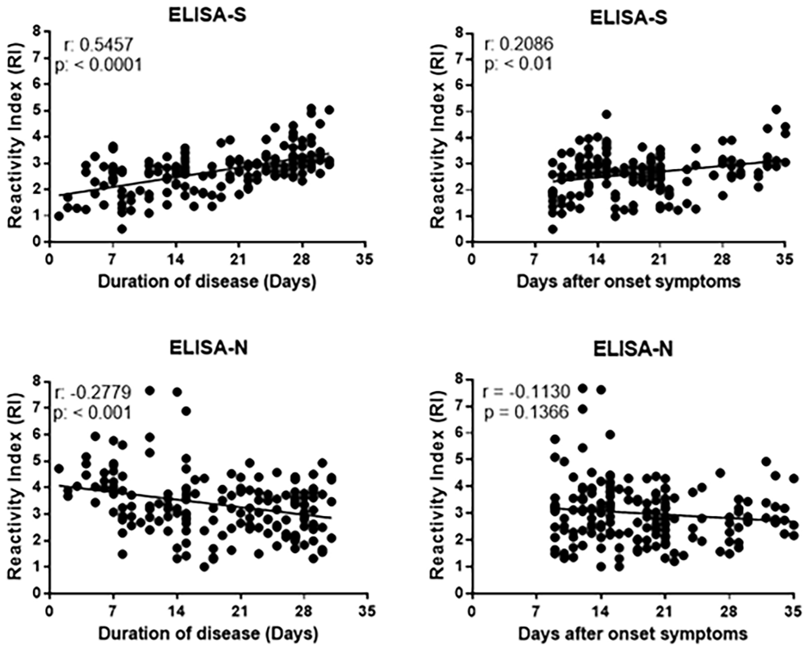


**Supplementary Figure S8** – Associations between levels of IgG anti-S-RBD and anti-N and days with COVID-19 symptoms or days post-symptom onset. At left: the reactivity index (RI) values from ELISA-S and ELISA-N and days of disease duration (one to 31 days); at right: The RI values from ELISA-S and ELISA-N and days post-symptoms onset (nine to 35 days). Data represent patients with confirmed COVID-19, n = 175. Spearman correlation test. Duration of disease: < 7 days n = 23; 8–15 days n = 56; > 15 days n = 96; Days after onset of symptoms: < 15 days n = 78; 16 –21 days n = 51; > 21 days n = 46.

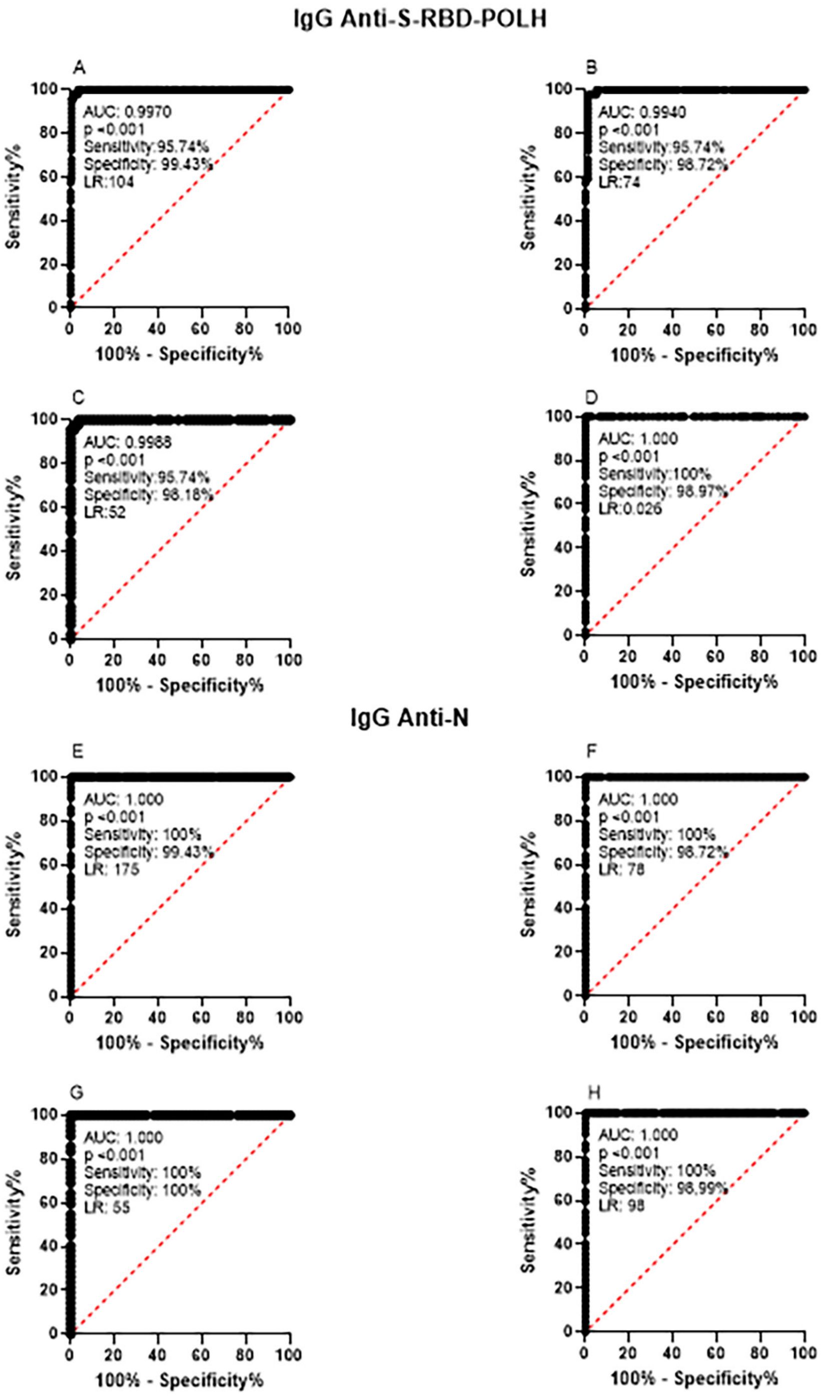


**Supplementary Figure S9** – ROC analyses of data from enzyme-linked immunosorbent assay-S (ELISA-S) and ELISA-N according to days post-symptom onset: (A) and (E) Data from 175 RT-qPCR positive patients and controls (A, n = 47; E, n = 42 controls; sera collected before pandemic); (B) and (F) Data from ≤ 7 days since disease onset, n = 23 patients; (C) and (G) Data from 8–15 days, n = 56 patients; (D) and (H) Data from > 15 days, n = 96 patients. Sensitivity, specificity, Area under the curve (AUC), Positive Likelihood ratio (LR), and p values; (D) as specificity was 100%, it is shown the negative LR (NLR).

**Supplementary Table S1** - Characteristics of participants serologically evaluated to COVID-19 in Goiania city, Goias State, Brazil, 2020-2024: seroepidemiological survey (1st cohort), vaccination and hybrid immunity (2nd and 3rd cohorts).

| | Uninfected (1st Cohort) 2020 (n = 200) | Patients (1st Cohort) 2020 (n = 100)* | Patients (1st Cohort) 2021 (n = 100)* | 2nd Cohort 2021-2022 (n=58) | 3rd Cohort 2023-2024 (n=40)** |
|---|---|---|---|---|---|
| **Sex** | | | | | |
| Male | 87 (43.5%) | 44 (44.0 %) | 36 (36.0 %) | 45 (77.6%) | 25 (62.5%) |
| Female | 113 (56.5%) | 56 (56.0 %) | 64 (64.0 %) | 13 (22.4%) | 15 (37.6%) |
| **Age** | 46 (20 - 67) | 32 (9 – 77) | 32 (9 – 77) | 38 (36 - 40) | 33 (30 - 41) |
| **Duration of the disease (Days with symptoms)** | --- | 7 (1 – 22) | 9 (4 – 31) | - | 4 (1 – 10) |
| **From onset of symptoms to serum collection (Days)** | --- | 18 (9 – 24) | 21 (12 – 35) | - | 15 (10 - 20) |
| **Interval between the COVID-19 diagnosis and the administration of the 1st vaccine dose** | -- | -- | -- | 3 months (2 – 5) | 60 days (60-80) |
| **Groups - Post-COVID Infection-naïve** | -- | -- | -- | 17 (29.4%)<br>41 (70.6%) | -- |
| **Confirmed Diagnosis** | | | | | |
| Real time RT-qPCR | 182 T (1P/182, 0,5%)<br>18 NT (18/200, 9%) | 85 T (85P/100, 85 %)<br>15 NT (15/100, 15%) | 89 T (89P/100, 89 %)<br>11 NT (11/100, 11%) | 58 T (58/58, 100%) | 40 T (40/40, 100%) |
| Rapid Test (LFIA) | 0 (0/200, 0%) | 100 SR (100/100, 100%) | 100 SR (100/100, 100%) | - | - |
| Chemiluminescence (CLIA) | 1 SR (1/200, 0.5 %)[a]<br>199 SNR (199/200, 99,5%) | 97 SR (97/100, 97 %)<br>3 SNR (3/100 , 3%)[b] | 92 SR (92/100, 92 %)<br>8 SNR (8/100, 8%)[c] | - | - |

Serum samples were collected between June and July 2020 (1st cohort); *All patients reported mild symptoms generally cough, fever, headache, runny nose, sore throat and moderate symptoms such as memory loss and difficulty breathing; [a]Positive sample for chemiluminescence and real time RT-PCR; [b]Non-reactive serum samples (n = 3/3) positive for chemiluminescence and RT-qPCR; [c]Five samples (5/8) real time RT-qPCR positive. The results were presented in absolute values, percentage and median (minimum and maximum); T = samples tested for real time RT-qPCR; P = positive samples; NT = samples not tested for real time RT-PCR; SR = reagent sera; SNR = non-reactive sera. Real time RT-PCR in nasopharynx samples. 1st = first cohort (2020-2021); 2nd = second cohort (2021-2022); 3rd = third cohort (2023-2024); **All patients had received one of the available vaccine regimens available: CoronaVac (Sinovac Biotech), ChAdOx1 nCoV-19 (AstraZeneca), Ad26.COV2.S/JNJ-78436735 (Janssen), or Tozinameran/Comirnaty (Pfizer-BioNTech).
